# Author Correction: Identification of a dual orange/far-red and blue light photoreceptor from an oceanic green picoplankton

**DOI:** 10.1038/s41467-021-27712-8

**Published:** 2021-12-16

**Authors:** Yuko Makita, Shigekatsu Suzuki, Keiji Fushimi, Setsuko Shimada, Aya Suehisa, Manami Hirata, Tomoko Kuriyama, Yukio Kurihara, Hidefumi Hamasaki, Emiko Okubo-Kurihara, Kazutoshi Yoshitake, Tsuyoshi Watanabe, Masaaki Sakuta, Takashi Gojobori, Tomoko Sakami, Rei Narikawa, Haruyo Yamaguchi, Masanobu Kawachi, Minami Matsui

**Affiliations:** 1 Synthetic Genomics Research Group, RIKEN Center for Sustainable Resource Science, Yokohama, Japan; 2 Biodiversity Division, National Institute for Environmental Studies, Tsukuba, Japan; 3 Graduate School of Integrated Science and Technology, Shizuoka University, Shizuoka, Japan; 4 Research Institute of Green Science and Technology, Shizuoka University, Shizuoka, Japan; 5 Core Research for Evolutional Science and Technology, Japan Science and Technology Agency, Saitama, Japan; 6 Yokohama City University, Kihara Institute for Biological Research, Yokohama, Japan; 7 Graduate School of Agricultural and Life Sciences, The University of Tokyo, Tokyo, Japan; 8 Fisheries Resources Institute, Japan Fisheries Research and Education Agency, Kushiro, Hokkaido Japan; 9 Department of Biological Sciences, Ochanomizu University, Tokyo, Japan; 10 Computational Bioscience Research Center, King Abdullah University of Science and Technology, Thuwal, Kingdom of Saudi Arabia; 11 Fisheries Resources Institute, Japan Fisheries Research and Education Agency, Minami-ise, Mie Japan; 12 Department of Biological Sciences, Graduate School of Science, Tokyo Metropolitan University, Tokyo, Japan

**Keywords:** Genome, Light responses, Plant molecular biology

Correction to: *Nature Communications* 10.1038/s41467-021-23741-5, published online 16 June 2021.

In the original version of this Article, the label of the domain structure that has 612 amino acids presented in Fig. 1a was given incorrectly as “AtCRY1”. The correct label should be written as “AtCRY2”. The display of Fig. 1 has been updated in both the PDF and HTML versions of the Article.

