# Supplementary material for: Identification of a dual orange/far-red and blue light photoreceptor from an oceanic green picoplankton
Source: Nat Commun. 2021 Jun 16;12:3593. doi: 10.1038/s41467-021-23741-5 (PMC8209157; doi:10.1038/s41467-021-23741-5)
Supplement: Supplementary file 1 — Supplementary Information [file 41467_2021_23741_MOESM1_ESM.pdf]

Identification of a dual orange/far-red and blue light photoreceptor from an oceanic green picoplankton

Makita *et al.*

Supplementary Note 1. Species composition of prasinophytes in Sendai Bay and the western subarctic Pacific Ocean

To elucidate the species composition of prasinophytes in the ocean, we analyzed parts of the metagenomic data (May 2012 to May 2013) in Sendai Bay (stations C5 and C12) and the western subarctic Pacific Ocean (stations A4 and A21) (Supplementary Fig. 1). These metagenic data cover varying depths (1 m to 50 m from the surface) and a wide range of ocean environments (coast to open ocean). In Sendai Bay (stations C5 and C12), the *P. provasolii* reads were 4–29% (C5) and 4–56% (C12) of the total prasinophyte reads. In particular, *P. provasolii* was dominant in June in the subsurface chlorophyll maximum (SCM; C5: 20 m, C12: 30 m). In the western subarctic Pacific Ocean (stations A4 and A21), the *P. provasolii* reads were 8–30% (A4) and 3–34% (A21). For station A4, *P. provasolii* was the year-round subdominant species on the surface and the SCM. For station A21, *P. provasolii* was dominant in July in the SCM (30 m). These data show that *P. provasolii* is a major species at various depths, in different environments, and during all seasons.

Supplementary Note 2. Genome completeness

The reads were assembled into 43 scaffolds with no gaps. Our genome size estimation based on k-mer counts showed that the genome is 19.6 Mbp, which is similar to the assembly size (22.7 Mbp) (Fig. 1c). All of the 43 scaffolds possessed telomere-like structures ($[5'\text{-GGGTTA-}3']_n$) at both termini. To confirm the number of chromosomes, we performed PFGE analysis (Supplementary Fig. 9a, b). Unfortunately, however, not all the chromosome bands could be resolved. Some showed clear, strong signals, showing that multiple bands overlapped. When the band pattern is predicted based on the scaffold size, it is mostly consistent with that observed by PFGE analysis. However, for small chromosomes less than 225 kbp, the sizes are larger than those of the corresponding scaffolds. This is probably because the small scaffolds are affected by incomplete telomere sequences more than larger scaffolds. These results suggest that the scaffolds correspond to chromosomes and the genome is complete.

Supplementary Method 1. Metagenomic analyses

To elucidate the composition of prasinophytes in the ocean, we analyzed the metagenomic data collected from March 2012 to March 2013 in Sendai Bay (stations C5 and C12) and the western subarctic Pacific Ocean (stations A4 and A21). The data were downloaded from the Ocean Monitoring Database (http://marine-meta.healthscience.sci.waseda.ac.jp/crest/). The genome dataset for prasinophytes was composed of 12 prasinophyte genomes that were available: *P. provasolii*, *Ostreococcus tauri* RCC4221[1], *O. tauri* RCC1115[2], *O. 'lucimarinus'*[3], *O. mediterraneus*[4], *Micromonas commoda* RCC299[5], *M. pusilla* CCMP1545[5], *Bathycoccus prasinos*[6], *Cymbomonas tetramitiformis*[7], *Chloropicon primus*[8], *Picocystis* sp. strain ML[9], and *Tetraselmis striata*[10]. The metagenomic reads were mapped on the genome dataset using segemehl 0.3.4[11] with similarity >80%. The hit strategy was best hit, which aligns only the most similar reads to the reference under the similarity criteria. The read counts were normalized by the assembly size.

Supplementary Method 2. Pulsed-field gel electrophoresis

We performed pulsed-field gel electrophoresis (PFGE) following Tanifuji *et al.*[12] with slight modifications. NIES-2893 cells were cultivated for ~20 days in IMK medium. The cells were collected by gentle centrifugation and washed with IMK once. The cell pellet was resuspended in envelope buffer (10 mM Tris–HCl at pH 8.0, 100 mM EDTA, 200 mM NaCl, and 0.8% low-melting agarose) to a final cell concentration of $\sim 1.0 \times 10^{10}$ cells/mL. The cell plugs were incubated in sarkosyl buffer (10 mM Tris–HCl at pH8.0, 400 mM EDTA, 1% N-lauryl sarkosyl and 1 mg/mL proteinase K) at 50 ºC for 24 h. Subsequently, the plugs were rinsed with wash buffer (10 mM Tris– HCl at pH8.0, 400 mM EDTA) three times. The plugs were incubated in SDS buffer (10 mM Tris–HCl at pH8.0, 100 mM EDTA, 1% SDS, 1 mg/mL proteinase K) at 50 ºC for 24 h. The digested plugs were again rinsed with wash buffer three times and stored in TE buffer at 4 ºC. Electrophoresis was performed using CHEF DR-II (Bio-Rad Laboratories, Hercules, CA, USA) with a pulse time of 5.3–106 s for 27 h at 185 V in 0.5x TBE buffer on a 1% agarose gel. The gel was stained with ethidium bromide solution. Images of the bands were captured by reversing black and white using ImageJ[13].

Supplementary Method 3. Genome size estimation

Genome size estimation was performed based on k-mer counts. k-mer counting was performed using Jellyfish 2.1.4[14], which counted canonical 25-mers, with forward reads of the paired-end reads. The k-mer graphs were analyzed using R scripts (https://www.r-project.org/).

Supplementary Method 4. Phylogenetic analysis of 105 proteins

To elucidate the phylogenetic position of *P. provasolii* in Viridiplantae, we performed phylogenetic analysis using 105 nuclear-encoded single-copy proteins. The dataset was composed of 21 species including *P. provasolii*. Four species in Streptophyta were used as an outgroup. The highly conserved orthologs were searched using the reciprocal best-hit analyses with a cut-off: similarity >60% and HSP coverage >40%. Proteins were downloaded from Phycocosm[15] or MMETSP[16]. The 105 proteins were shared by all of the species. The sequences were aligned using MAFFT v7.453[17] with an auto option. The alignments were trimmed using trimAl v1.4.rev15[18] with the option automated1. The trimmed dataset contained 41,023 amino acids. The model test was performed using ModelTest-NG v0.1.5[19]. Maximum likelihood analysis was performed using RAxML-NG v0.9.0[20] with 200 bootstrap replicates. For Bayesian analysis, MrBayes v3.2.7a was used with WAG + GAMMA + I substitutional model, which was tested using ModelTest-NG. Bayesian inference consisted of 1,000,000 generations with sampling at every 1,000 generations using the four Metropolis-coupled Markov chain Monte Carlo (MCMCMC) simulations. Two separate runs were performed, and the convergence was assessed by the average standard deviation of split frequencies (ASDSF) falling below 0.01. Bayesian posterior probabilities (BPP) were calculated from the majority rule consensus of the trees sampled after the initial 250 burn-in trees.

Supplementary Method 5. Comparison of gene content among prasinophytes

We compared orthogroups among representative prasinophytes: *P. provasolii*, *C. primus*, *M. commoda*, *M. pusilla*, *O. tauri*, *O. 'lucimarinus'*, and *B. prasinos*. The orthogroups were searched using OrthoFinder[21]. Orthogroups of mamiellophyceans (*Micromonas*, *Ostreococcus*, and *Bathycoccus*) were merged.

Supplementary Method 6. Phylogenetic analyses for *Lhcp*

A blastp search was carried out using homologous proteins of Lhcp of *P. provasolii* to the proteins of prasinophytes in the PhycoCosm database (https://phycocosm.jgi.doe.gov/phycocosm/home)[22]. The sequences were aligned using MAFFT v7.427[17] with the linsi option and manually trimmed. The ML tree was inferred using IQ-TREE v1.6.12[23] with 100 non-parametric bootstrap replicates.

Supplementary Method 7. Quantitative RT-PCR analysis

cDNA was synthesized using QuantiTect Reverse Transcription Kits (QIAGEN) from total RNA of *P. provasolii*, according to the manufacturer's instructions. Quantitative real-time PCR was performed using THUNDERBIRD SYBR qPCR Mix (TOYOBO) and CFX Connect (Bio-Rad). The primers used for qRT - PCR were shown in the Supplementary Table 2.

Supplementary Method 8. Generating transgenic *Arabidopsis* for complementation assay

PpDUC1 cDNA with an HA sequence and a stop codon was amplified from *P. provasolii* genomic DNA using the primers (Supplementary Table 2). It was firstly cloned into pENTR/D-TOPO (Invitrogen) and subsequently into the binary vector pMDC32 [24] containing a *CaMV 35S* promoter using the Gateway strategy (Thermo Fisher Scientific). The resulting *CaMV 35S-PpDUC1* constructs were transformed into *phyB* (Salk_022035) and *cry2-1*[25] mutants via the floral dipping method[26]. The transgenic plants were screened on 1/2 MS medium containing 25 mg/L hygromycin.

Supplementary Method 9. Measurement of hypocotyl length of transgenic *Arabidopsis*

For measurement of hypocotyls, sterilized seeds were sown on GM plates, cold treated at 4 ℃ for three days in the dark, and then exposed to white light for 6 hours to enhance germination. Following the white light treatment, plants were grown at 22℃ under continuous blue light (peak 447 nm, half bandwidth of 20 nm), orange light (peak 596 nm, half bandwidth of 20 nm), red light (peak 650 nm, half bandwidth of 25 nm) and in the dark. Seven-day-old seedlings were photographed, and the hypocotyl lengths were measured using ImageJ software (http://rsb.info.nih.gov/ij/). The seedlings were examined for each light condition.

Supplementary Method 10. Visualization and analysis of molecular structure

Molecular graphics of a crystal structure of 15*Z*–phycocyanobilin (PCB)-bound SyCph1 (PDB ID: 2VEA)[27] were generated by UCSF Chimera software[28].

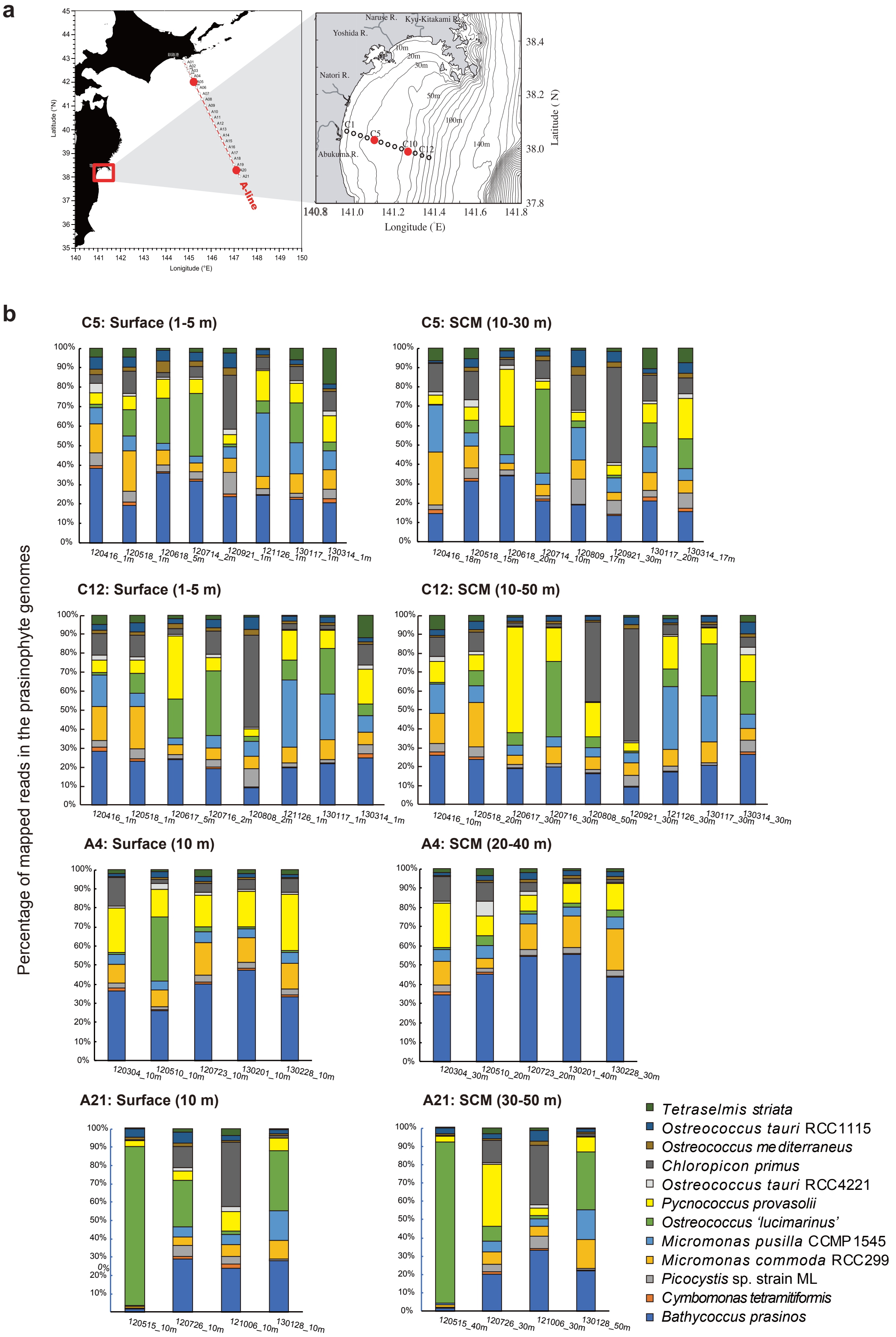
a
Naruse R.
Kyu-Kitakami R.
Yoshida R.
Natori R.
Abukuma R.
C1
C5
C10
C12
A-line
Latitude (°N)
Longitude (°E)
Latitude (N)
Longitude (E)
b
C5: Surface (1-5 m)
C5: SCM (10-30 m)
C12: Surface (1-5 m)
C12: SCM (10-50 m)
A4: Surface (10 m)
A4: SCM (20-40 m)
A21: Surface (10 m)
A21: SCM (30-50 m)
Percentage of mapped reads in the prasinophyte genomes
Tetraselmis striata
Ostreococcus tauri RCC1115
Ostreococcus mediterraneus
Chloropicon primus
Ostreococcus tauri RCC4221
Pycnococcus provasolii
Ostreococcus 'lucimarinus'
Micromonas pusilla CCMP1545
Micromonas commoda RCC299
Picocystis sp. strain ML
Cymbomonas tetramitiformis
Bathycoccus prasinos

Supplementary Fig. 1. Metagenome sampling locations and species composition of prasinophytes in Sendai Bay and the western subarctic Pacific Ocean.

(a) Metagenome sampling locations (red dots). **(b)** We analyzed the metagenomic data collected from May 2012 to May 2013 in Sendai Bay (stations C5 and C12) and the western subarctic Pacific Ocean (stations A4 and A21). The data were downloaded from the Ocean Monitoring Database (http://marine-meta.healthscience.sci.waseda.ac.jp/crest/). Twelve strains with complete/draft genomes were used as references and are indicated in different colors. Sampling dates and depths are shown under each bar.

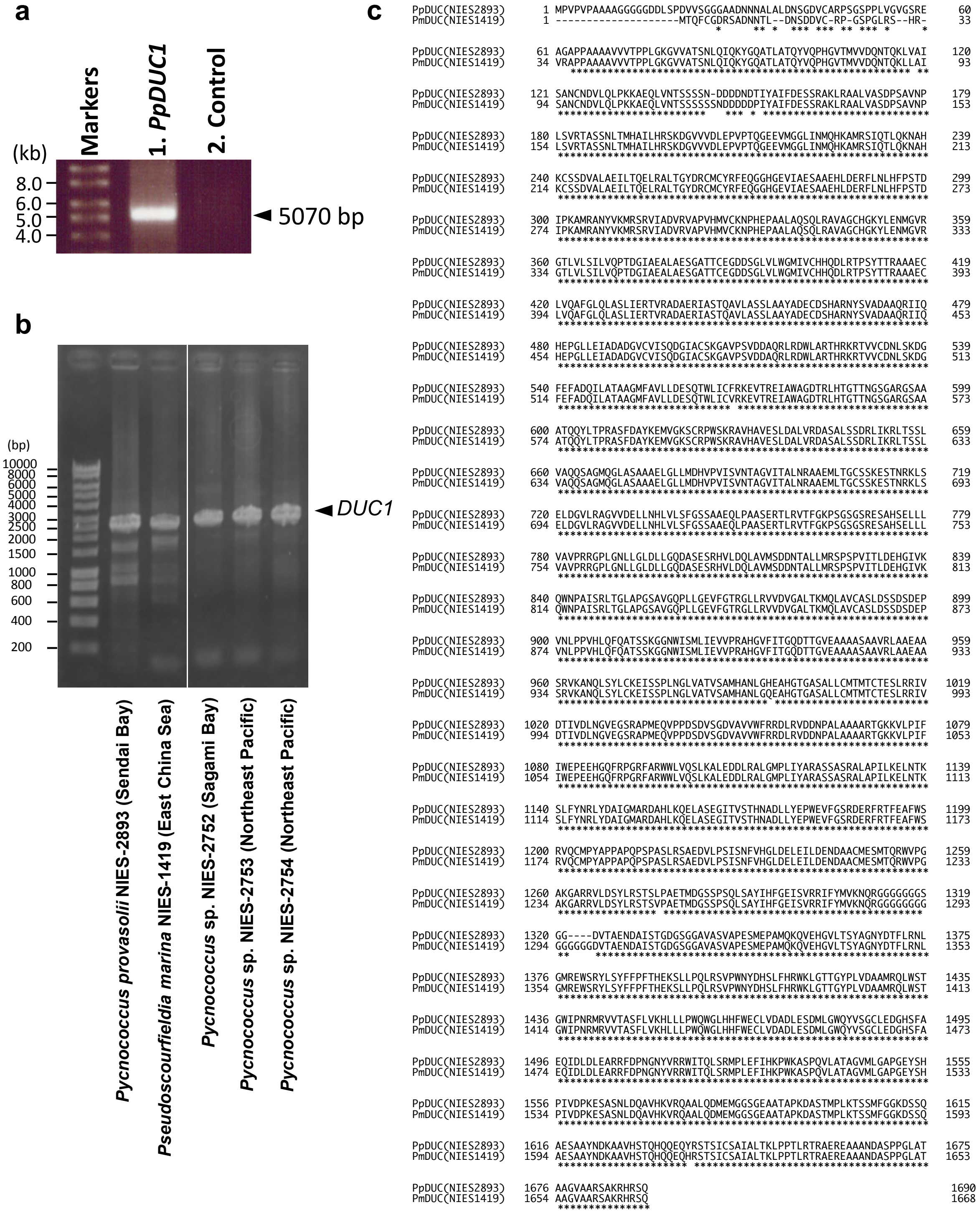

Supplementary Fig. 2. The expression of PpDUC1 and its genome sequences.

(a) A cDNA fragment was obtained by reverse transcription PCR (RT-PCR) from *P. provasolii* using specific primers to amplify the full-length coding sequence of the *PpDUC* gene. Lane M:

molecular markers; Lane 1: cDNA of the *PpDUC* gene was 5070 bp in length; Lane 2: Negative control was PCR amplification (without a reverse transcriptase reaction) from total RNA extracted from *P. provasolii*. Experiments were performed three times with similar results. **(b)** Detection of *DUC1* genes with PCR from four strains of Pycnococcus (NIES-2893, NIES-2752, NIES-2753, and NIES-2754) and *Pseudoscourfieldia marina* (NIES-1419) from the NIES culture collection. DNA was extracted using DNeasy Plant Mini Kits (Qiagen) and genomic PCR was performed with primers DUC_f2: 5'-ATGGAGCAAGCGTGCCGTGC-3' and DUC_r2: 5'-GGTGGACCACAGCTGGCGCA-3'. The PCR products were sequenced using the DUC_f2 primer. Sampling locations are shown in parentheses. Experiments were performed twice with similar band patterns. We checked the bands were *DUC1* by sequencing. **(c)** Multiple alignment of PpDUC1 (NIES-2893) and PmDUC1 (NIES-1419) genes. The source data of the gel blots in (a,b) are provided in the Source Data file.

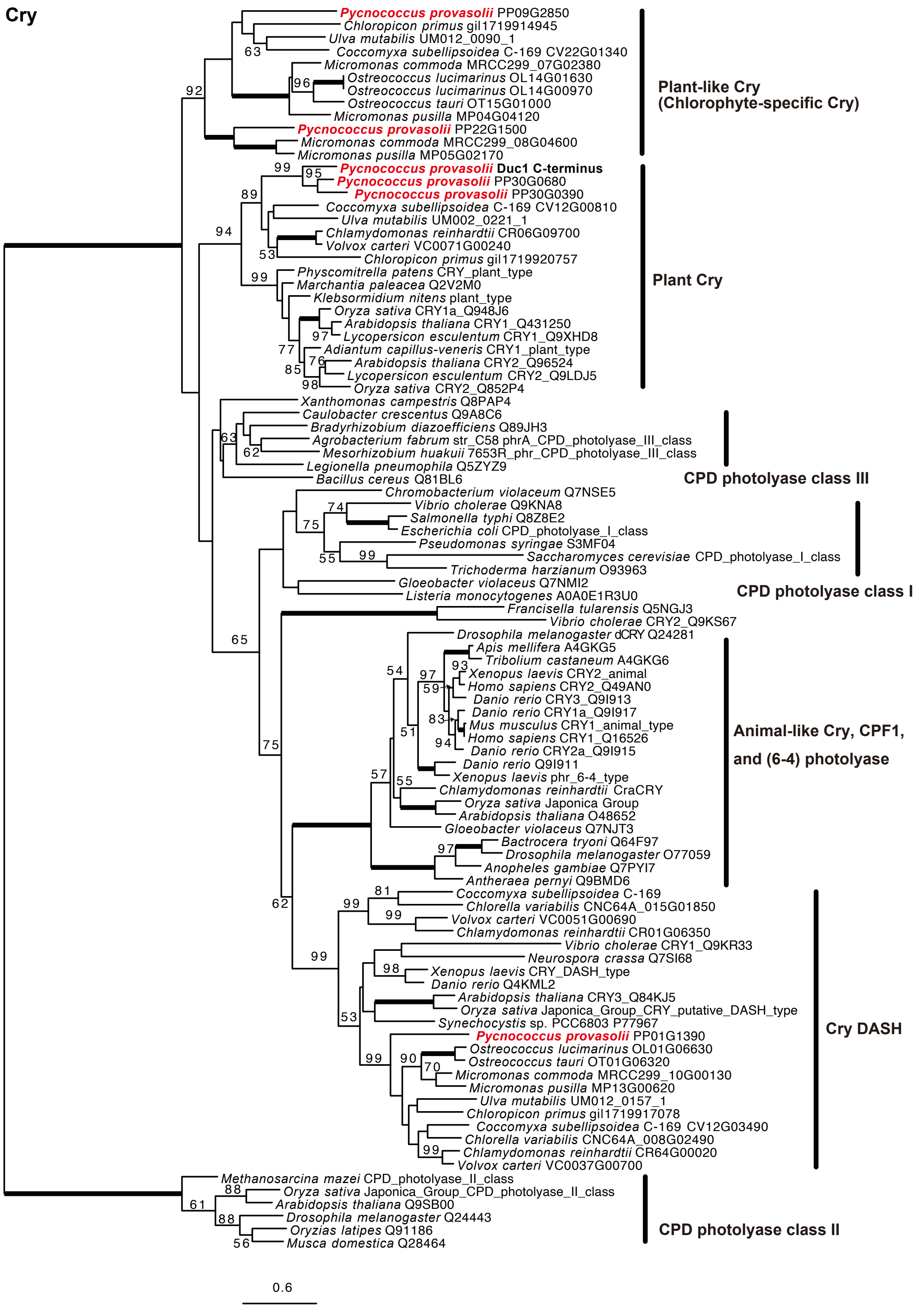
Cry
Pycnococcus provasolii PP09G2850
Chloropicon primus gi|1719914945
Ulva mutabilis UM012_0090_1
Coccomyxa subellipsoidea C-169 CV22G01340
Micromonas commoda MRCC299_07G02380
Ostreococcus lucimarinus OL14G01630
Ostreococcus lucimarinus OL14G00970
Ostreococcus tauri OT15G01000
Micromonas pusilla MP04G04120
Pycnococcus provasolii PP22G1500
Micromonas commoda MRCC299_08G04600
Micromonas pusilla MP05G02170
Pycnococcus provasolii Duc1 C-terminus
Pycnococcus provasolii PP30G0680
Pycnococcus provasolii PP30G0390
Coccomyxa subellipsoidea C-169 CV12G00810
Ulva mutabilis UM002_0221_1
Chlamydomonas reinhardtii CR06G09700
Volvox carteri VC0071G00240
Chloropicon primus gi|1719920757
Physcomitrella patens CRY_plant_type
Marchantia paleacea Q2V2M0
Klebsormidium nitens plant_type
Oryza sativa CRY1a_Q948J6
Arabidopsis thaliana CRY1_Q431250
Lycopersicon esculentum CRY1_Q9XHD8
Adiantum capillus-veneris CRY1_plant_type
Arabidopsis thaliana CRY2_Q96524
Lycopersicon esculentum CRY2_Q9LDJ5
Oryza sativa CRY2_Q852P4
Xanthomonas campestris Q8PAP4
Caulobacter crescentus Q9A8C6
Bradyrhizobium diazoefficiens Q89JH3
Agrobacterium fabrum str_C58 phrA_CPD_photolyase_III_class
Mesorhizobium huakuii 7653R_phr_CPD_photolyase_III_class
Legionella pneumophila Q5ZYZ9
Bacillus cereus Q81BL6
Chromobacterium violaceum Q7NSE5
Vibrio cholerae Q9KNA8
Salmonella typhi Q8Z8E2
Escherichia coli CPD_photolyase_I_class
Pseudomonas syringae S3MF04
Saccharomyces cerevisiae CPD_photolyase_I_class
Trichoderma harzianum O93963
Gloeobacter violaceus Q7NMI2
Listeria monocytogenes A0A0E1R3U0
Francisella tularensis Q5NGJ3
Vibrio cholerae CRY2_Q9KS67
Drosophila melanogaster dCRY Q24281
Apis mellifera A4GKG5
Tribolium castaneum A4GKG6
Xenopus laevis CRY2_animal
Homo sapiens CRY2_Q49AN0
Danio rerio CRY3_Q9I913
Danio rerio CRY1a_Q9I917
Mus musculus CRY1_animal_type
Homo sapiens CRY1_Q16526
Danio rerio CRY2a_Q9I915
Danio rerio Q9I911
Xenopus laevis phr_6-4_type
Chlamydomonas reinhardtii CraCRY
Oryza sativa Japonica Group
Arabidopsis thaliana O48652
Gloeobacter violaceus Q7NJT3
Bactrocera tryoni Q64F97
Drosophila melanogaster O77059
Anopheles gambiae Q7PYI7
Antheraea pernyi Q9BMD6
Coccomyxa subellipsoidea C-169
Chlorella variabilis CNC64A_015G01850
Volvox carteri VC0051G00690
Chlamydomonas reinhardtii CR01G06350
Vibrio cholerae CRY1_Q9KR33
Neurospora crassa Q7SI68
Xenopus laevis CRY_DASH_type
Danio rerio Q4KML2
Arabidopsis thaliana CRY3_Q84KJ5
Oryza sativa Japonica_Group_CRY_putative_DASH_type
Synechocystis sp. PCC6803 P77967
Pycnococcus provasolii PP01G1390
Ostreococcus lucimarinus OL01G06630
Ostreococcus tauri OT01G06320
Micromonas commoda MRCC299_10G00130
Micromonas pusilla MP13G00620
Ulva mutabilis UM012_0157_1
Chloropicon primus gi|1719917078
Coccomyxa subellipsoidea C-169 CV12G03490
Chlorella variabilis CNC64A_008G02490
Chlamydomonas reinhardtii CR64G00020
Volvox carteri VC0037G00700
Methanosarcina mazei CPD_photolyase_II_class
Oryza sativa Japonica_Group_CPD_photolyase_II_class
Arabidopsis thaliana Q9SB00
Drosophila melanogaster Q24443
Oryzias latipes Q91186
Musca domestica Q28464
Plant-like Cry
(Chlorophyte-specific Cry)
Plant Cry
CPD photolyase class III
CPD photolyase class I
Animal-like Cry, CPF1,
and (6-4) photolyase
Cry DASH
CPD photolyase class II
0.6

Supplementary Fig. 3. The maximum likelihood phylogenetic tree of CRY proteins.

The dataset was composed of CRY proteins (353 amino acids) of 96 OTUs including the C-terminus of Duc1 of *P. provasolii*. The ML tree was inferred using RAxML-NG v. 0.9.0 with LG + I + G4 substitution model, which was tested using ModelTest-NG 0.1.5. Bootstrap analysis was replicated 200 times. Bootstrap supports (BPs) are represented on each node, and bold lines represent BP = 100. The C-terminus of Duc1 and the other 2 CRYs of *P. provasolii* are included in the plant Cry group, which is composed of CRYs of land plants (e.g. AtCRY1,2). The multiple alignment of this phylogenetic tree is provided in the Source Data file.

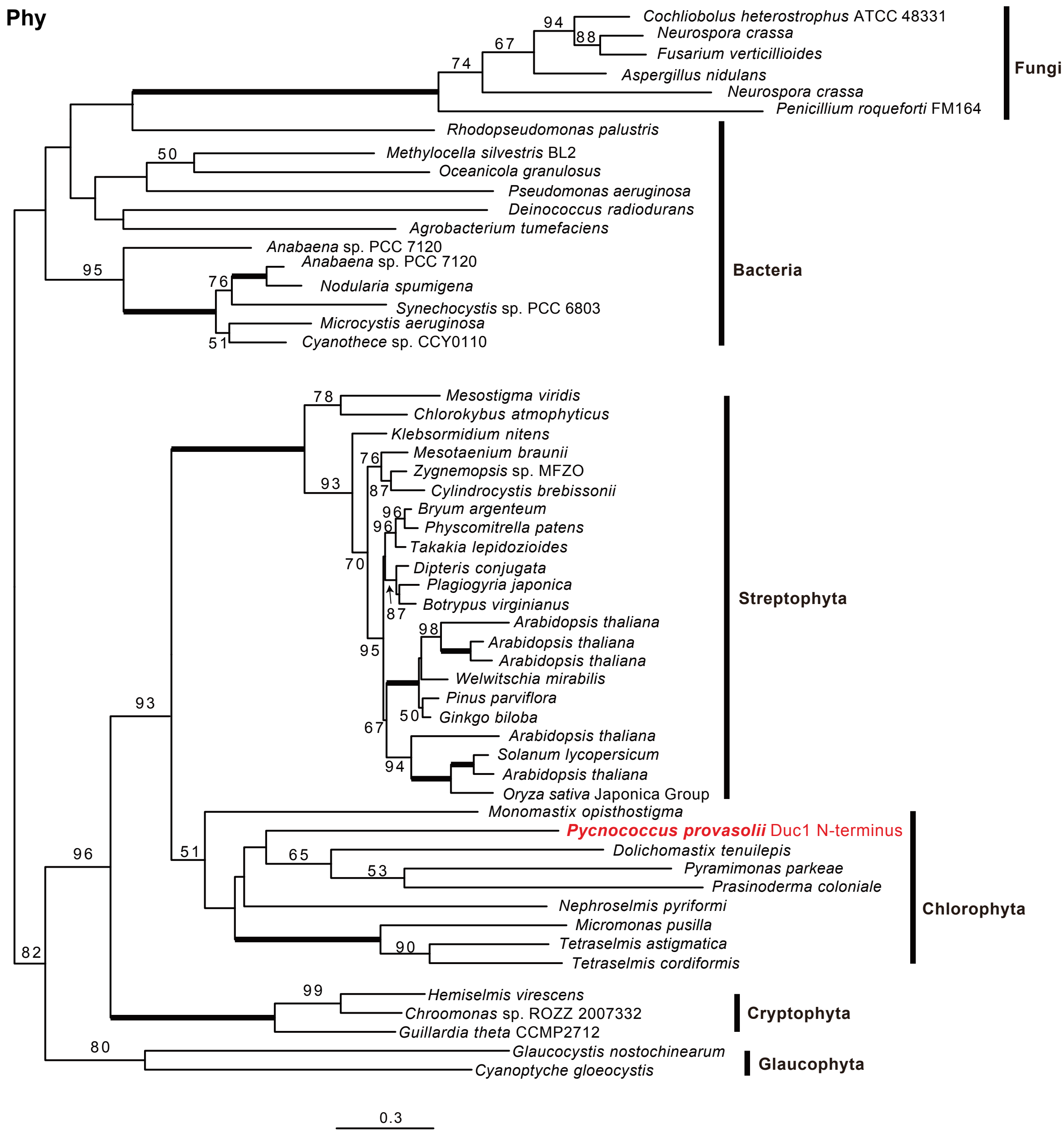

Supplementary Fig. 4. The maximum likelihood phylogenetic trees of PHY proteins.

The dataset was composed of PHY proteins (457 amino acids) of 54 OTUs including the N-terminus of Duc1 of *P. provasolii*. The ML tree was inferred using RAxML-NG v. 0.9.0 with LG + I + G4 substitution model, which was tested using ModelTest-NG 0.1.5. Bootstrap analysis was replicated 200 times. Bootstrap supports (BPs) are represented on each node, and bold lines represent BP = 100. The N-terminus of Duc1 is monophyletic with PHYs of prasinophytes, which is sister to those of streptophytes. The multiple alignment of this phylogenetic tree is provided in the Source Data file.

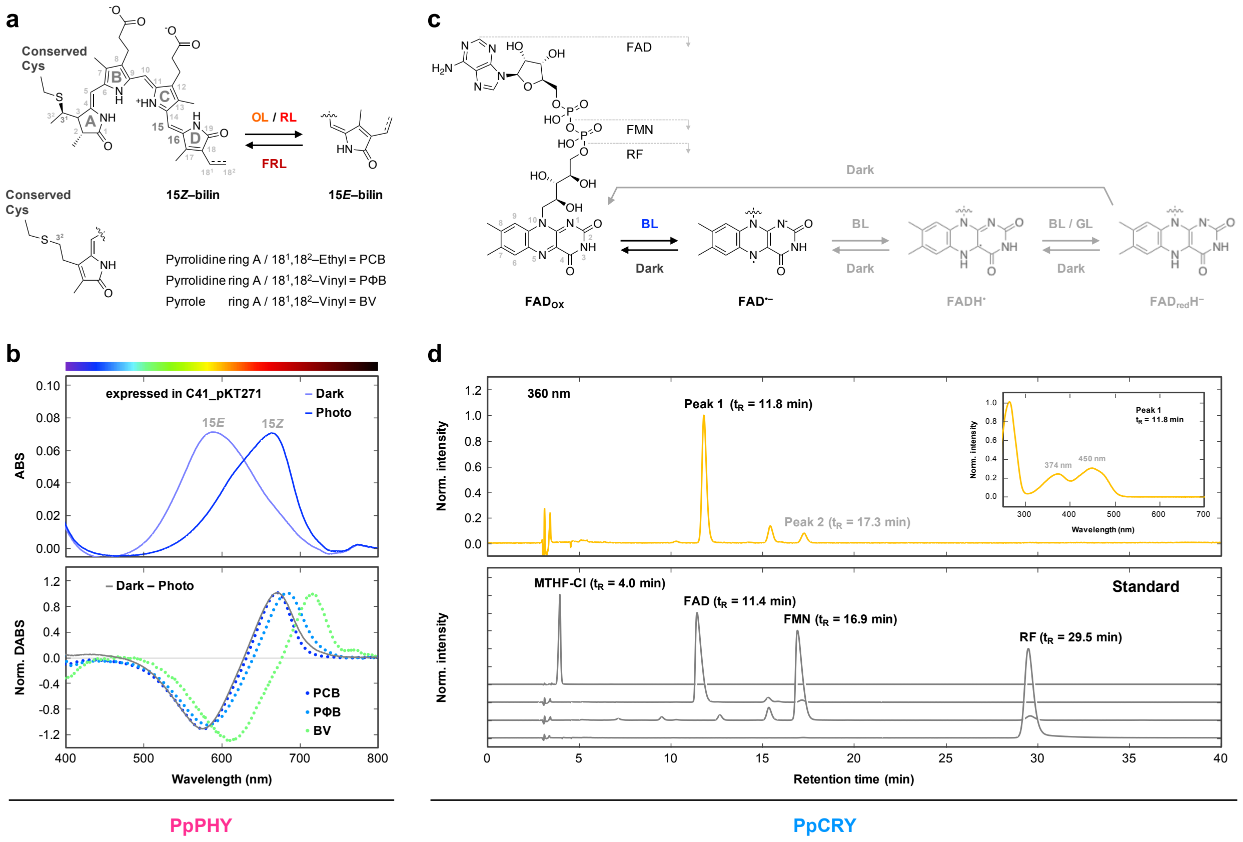

Supplementary Fig. 5. Assignment of chromophores incorporated into PpPHY and PpCRY.

(a) Photoreaction of bilins incorporated into phytochromes. Cyanobacterial and plant phytochromes incorporate PCB or PΦB via the GAF Cys, whereas bacterial and fungal ones incorporate BV via the N-terminal Cys. Light-induced *Z*/*E* isomerization of the C15=C16 double bond triggers a reversible photocycle between the dark state and the photoproduct state [29]. **(b)** Acid-denatured absorption spectra of the two states of PpPHY expressed in C41_pKT271. The normalized difference spectrum (dark state – photoproduct state) was compared with those of standard proteins [30,31] for binding chromophore assignment. **(c)** Photoreaction of flavin incorporated into cryptochromes. In many cases, cryptochromes non-covalently bind to FAD, but not to flavin mononucleotide (FMN) nor to riboflavin (RF), with 5,10-methenyltetrahydrofolate (MTHF) as a second chromophore. Some redox state forms of FAD, such as FAD_{OX}, $FAD^{\bullet-}$, $FADH^{\bullet}$ and $FAD_{red}H$, are observed during the reversible photocycle [32]. **(d)** HPLC chromatogram detecting chromophore(s) released from PpCRY expressed in C41_pG-KJE8, monitored at 360 nm. Through comparison with standard compounds, the main Peak 1 (t_R = 11.8 min) was assigned to FAD (inset shows its absorption spectrum). The minor Peak 2 (t_R = 17.3 min) could be assigned to FMN, but may be a degradation product from FAD produced during extraction.

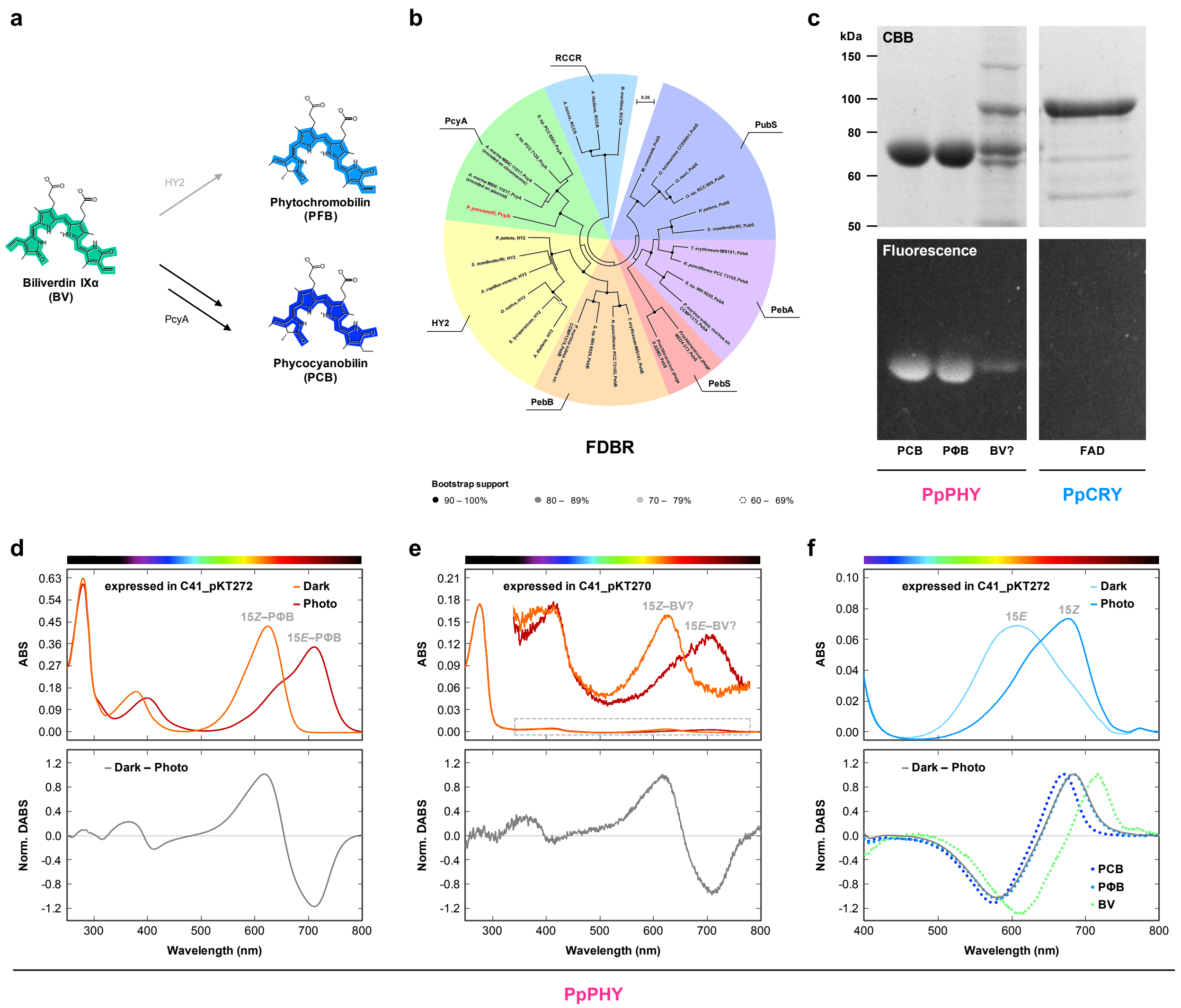

Supplementary Fig. 6. Specificity and capability of the chromophore incorporation.

(a) Biosynthesis of bilin chromophores. PCB and PΦB are produced from BV by 3*Z*-phycocyanobilin:ferredoxin oxidoreductase (PcyA) and 3*Z*-phytochromobilin:ferredoxin oxidoreductase (HY2), which are conserved in cyanobacteria, algae and land plants. π–conjugated systems are highlighted. **(b)** Phylogenetic tree of ferredoxin-dependent bilin reductases (FDBRs) including PcyA from *P. provasolii*. Each lineage is classified according to enzyme specificities. **(c)** SDS-PAGE analysis of His-tagged PpPHY (72.4 kDa) and GST-tagged PpCRY (102.2 kDa). The proteins and bound chromophores were detected by Coomassie Brilliant Blue (CBB) stain (upper) and zinc-dependent fluorescence imaging (lower). Similar results were obtained in two independent experiments. **(d,e)** Absorption spectra of the dark states (15*Z*–chromophores, Po form) and the photoproduct states (15*E*–chromophores, Pfr form) of PpPHY expressed in C41_pKT272 (D) and C41_pKT270 (E). Normalized difference spectra (dark states – photoproduct states) were calculated from these absorption spectra. **(f)** Acid-denatured absorption spectra of the two states of PpPHY expressed in C41_pKT272. The normalized difference spectrum (dark state – photoproduct state) was compared with those of

standard proteins[30,31] for binding chromophore assignment. A chromophore incorporated into PpPHY expressed in C41_pKT270 could not be assigned because of the low expression yield.

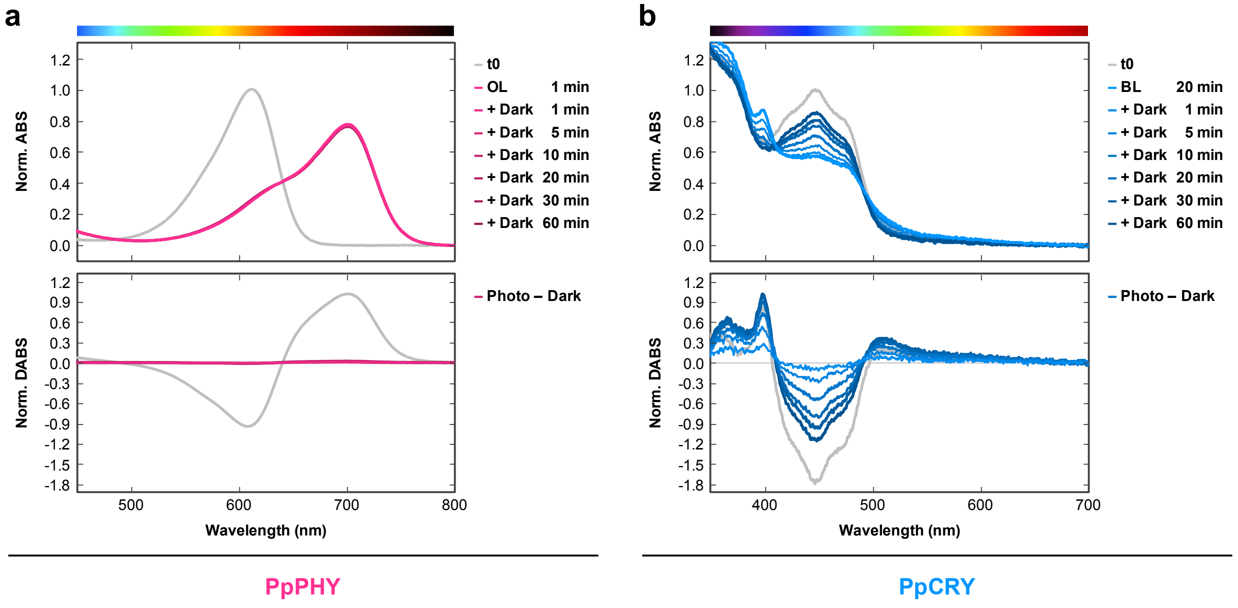

Supplementary Fig. 7. Transition processes of PpPHY and PpCRY in dark conditions.

(a) Normalized absorption spectra of PpPHY in the dark state (light gray) and the photoproduct state (magenta). The transition process from the photoproduct state (15*E*–PCB, Pfr form) to the dark state (15*Z*–PCB, Po form) was monitored at 1, 5, 10, 20, 30 and 60 min in dark conditions after orange-light irradiation (610 nm). **(b)** Normalized absorption spectra of PpCRY in the dark state (light gray) and the photoproduct state (cyan). The transition process from the photoproduct state ($FAD^{\bullet-}$, Puv form) to the dark state (FAD_{OX}, Pb form) was monitored in the same conditions after blue-light irradiation (450 nm). The normalized difference spectra (photoproduct state – dark state) of PpPHY and PpCRY were calculated from these absorption spectra.

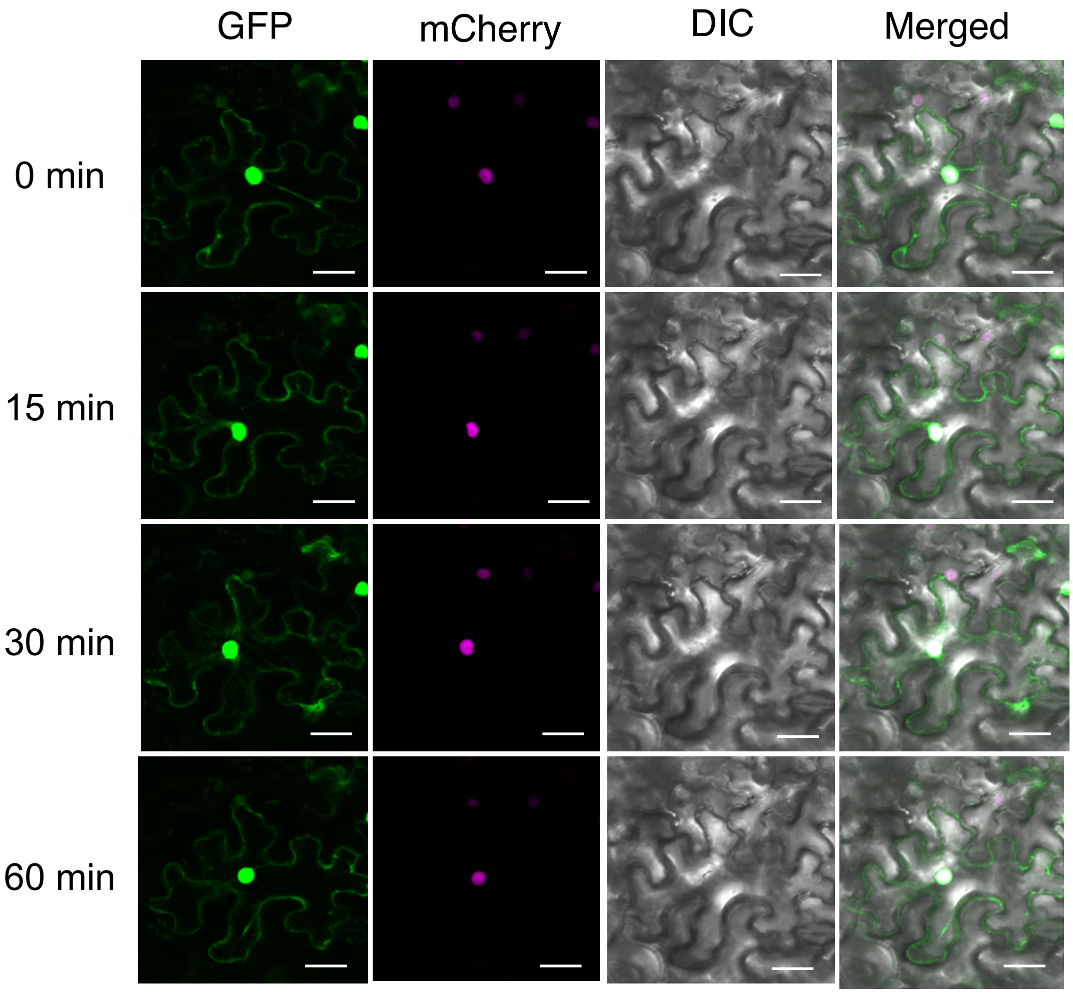

Supplementary Fig. 8. Localization of PpDUC1-GFP from the dark to light conditions.
Leaves of *N. benthamiana* plants were injected to induce expression of PpDUC1-GFP with HY5-mCherry. The plants were transferred to the dark. After 3 days, the leaves were exposed to white light and observed 0, 15, 30 and 60 min after exposure. DIC, differential interference contrast images; GFP, GFP fluorescence images; Merged, merged images of GFP, mCherry, and DIC images. Scale bar = 50 µm. Observations were performed three times with similar results.

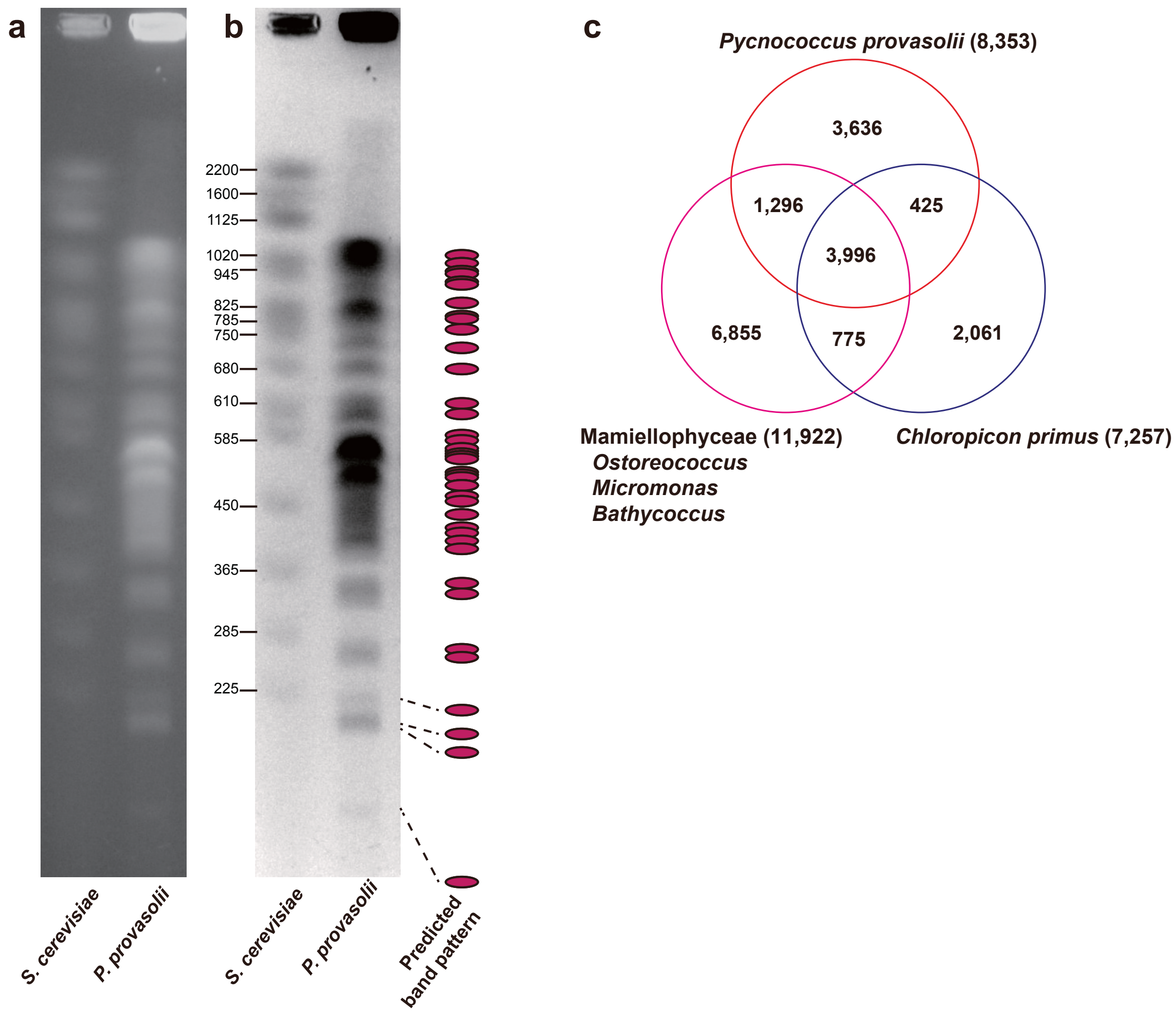

Supplementary Fig. 9. Chromosome composition of *P. provasolii* and orthogroups.

(a) Original image of the pulsed-field gel electrophoresis (PFGE). **(b)** Color-inverted image of the PFGE gel and the predicted band pattern based on scaffold sizes. We used the *S. cerevisiae* genome as a size marker. Putative correspondence between chromosomal bands (< 225 kbp) and the predicted bands is shown by dotted lines. We repeated the PFGE analyses three times with similar band patterns (a, b). **(c)** Venn diagram of orthogroups in *P. provasolii*, *C. primus*, and species in Mamiellophyceae (*Micromonas commoda*, *M. pusilla*, *O. tauri*, *O. 'lucimarinus'*, and *Batycoccus prasinos*). Orthogroups were identified using OrthoFinder 2.3.1021.

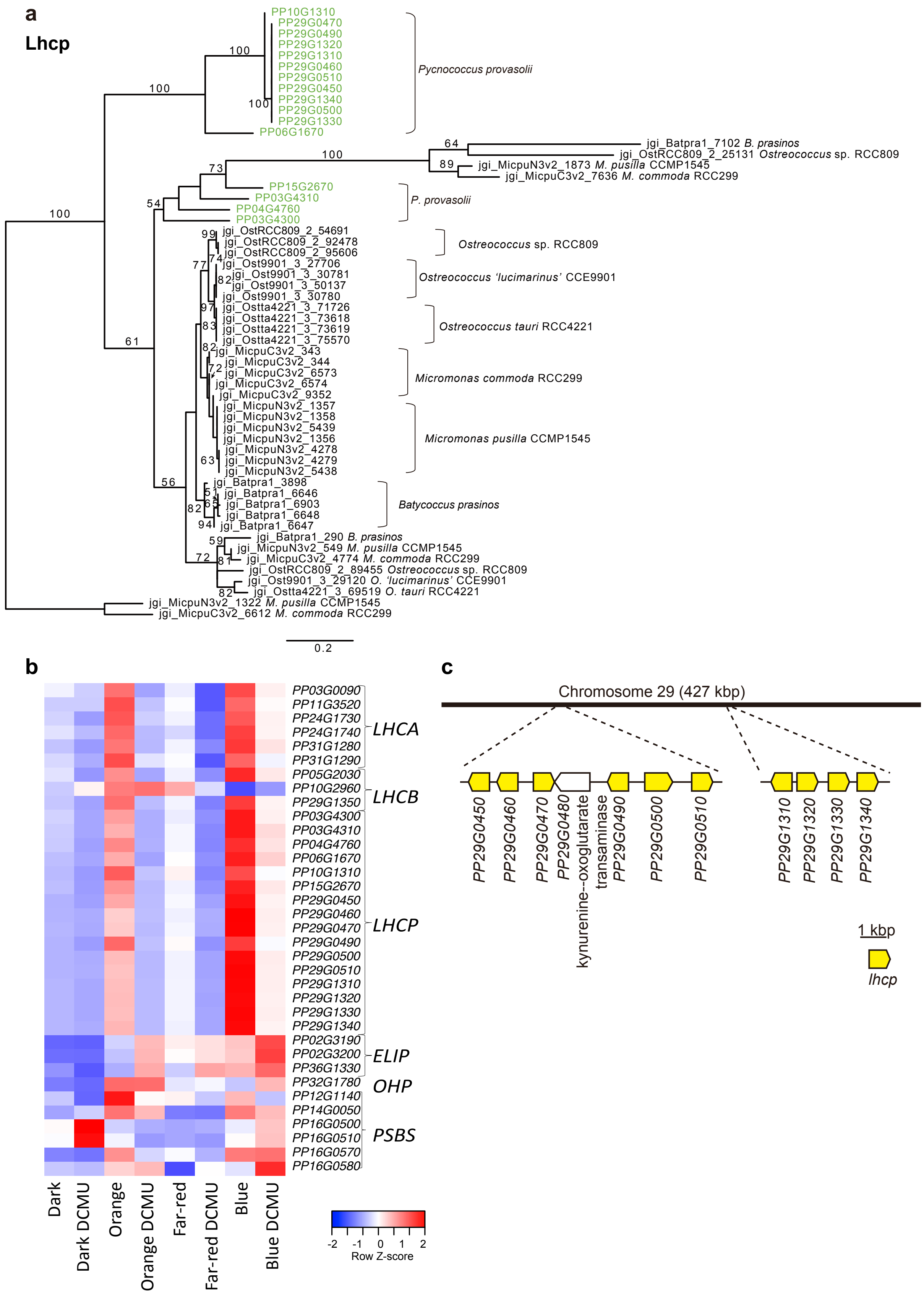
a
Lhcp
PP10G1310
PP29G0470
PP29G0490
PP29G1320
PP29G1310
PP29G0460
PP29G0510
PP29G0450
PP29G1340
PP29G0500
PP29G1330
PP06G1670
Pycnococcus provasolii
jgi_Batpra1_7102 B. prasinos
jgi_OstRCC809_2_25131 Ostreococcus sp. RCC809
jgi_MicpuN3v2_1873 M. pusilla CCMP1545
jgi_MicpuC3v2_7636 M. commoda RCC299
PP15G2670
PP03G4310
PP04G4760
PP03G4300
P. provasolii
jgi_OstRCC809_2_54691
jgi_OstRCC809_2_92478
jgi_OstRCC809_2_95606
Ostreococcus sp. RCC809
jgi_Ost9901_3_27706
jgi_Ost9901_3_30781
jgi_Ost9901_3_50137
jgi_Ost9901_3_30780
Ostreococcus 'lucimarinus' CCE9901
jgi_Ostta4221_3_71726
jgi_Ostta4221_3_73618
jgi_Ostta4221_3_73619
jgi_Ostta4221_3_75570
Ostreococcus tauri RCC4221
jgi_MicpuC3v2_343
jgi_MicpuC3v2_344
jgi_MicpuC3v2_6573
jgi_MicpuC3v2_6574
jgi_MicpuC3v2_9352
Micromonas commoda RCC299
jgi_MicpuN3v2_1357
jgi_MicpuN3v2_1358
jgi_MicpuN3v2_5439
jgi_MicpuN3v2_1356
jgi_MicpuN3v2_4278
jgi_MicpuN3v2_4279
jgi_MicpuN3v2_5438
Micromonas pusilla CCMP1545
jgi_Batpra1_3898
jgi_Batpra1_6646
jgi_Batpra1_6903
jgi_Batpra1_6648
jgi_Batpra1_6647
Batycoccus prasinos
jgi_Batpra1_290 B. prasinos
jgi_MicpuN3v2_549 M. pusilla CCMP1545
jgi_MicpuC3v2_4774 M. commoda RCC299
jgi_OstRCC809_2_89455 Ostreococcus sp. RCC809
jgi_Ost9901_3_29120 O. 'lucimarinus' CCE9901
jgi_Ostta4221_3_69519 O. tauri RCC4221
jgi_MicpuN3v2_1322 M. pusilla CCMP1545
jgi_MicpuC3v2_6612 M. commoda RCC299
0.2
b
PP03G0090
PP11G3520
PP24G1730
PP24G1740
PP31G1280
PP31G1290
LHCA
PP05G2030
PP10G2960
PP29G1350
LHCB
PP03G4300
PP03G4310
PP04G4760
PP06G1670
PP10G1310
PP15G2670
PP29G0450
PP29G0460
PP29G0470
PP29G0490
PP29G0500
PP29G0510
PP29G1310
PP29G1320
PP29G1330
PP29G1340
LHCP
PP02G3190
PP02G3200
PP36G1330
ELIP
PP32G1780
OHP
PP12G1140
PP14G0050
PP16G0500
PP16G0510
PP16G0570
PP16G0580
PSBS
Dark
Dark DCMU
Orange
Orange DCMU
Far-red
Far-red DCMU
Blue
Blue DCMU
-2 -1 0 1 2
Row Z-score
c
Chromosome 29 (427 kbp)
PP29G0450
PP29G0460
PP29G0470
PP29G0480
kynurenine--oxoglutarate transaminase
PP29G0490
PP29G0500
PP29G0510
PP29G1310
PP29G1320
PP29G1330
PP29G1340
1 kbp
lhcp

Supplementary Fig. 10. Prasinophyte-specific light-harvesting chlorophyll proteins.

(a) Unlooted ML tree of prasinophyte-specific light-harvesting chlorophyll proteins (Lhcp). The dataset was composed of 183 amino acids of 56 proteins, which were encoded in the prasinophyte genomes that were available in the PhycoCosm database. Green color indicates *P. provasolii* proteins. The multiple alignment of this phylogenetic tree is provided in the Source Data file. **(b)** Heatmap of RNA-seq expression z-score computed for *LHC* genes under monochromatic light conditions. **(c)** Genes for *Lhcp* in the chromosome 29. Ten genes for *Lhcp* (yellow) are tandemly encoded in two regions of chromosome 29.

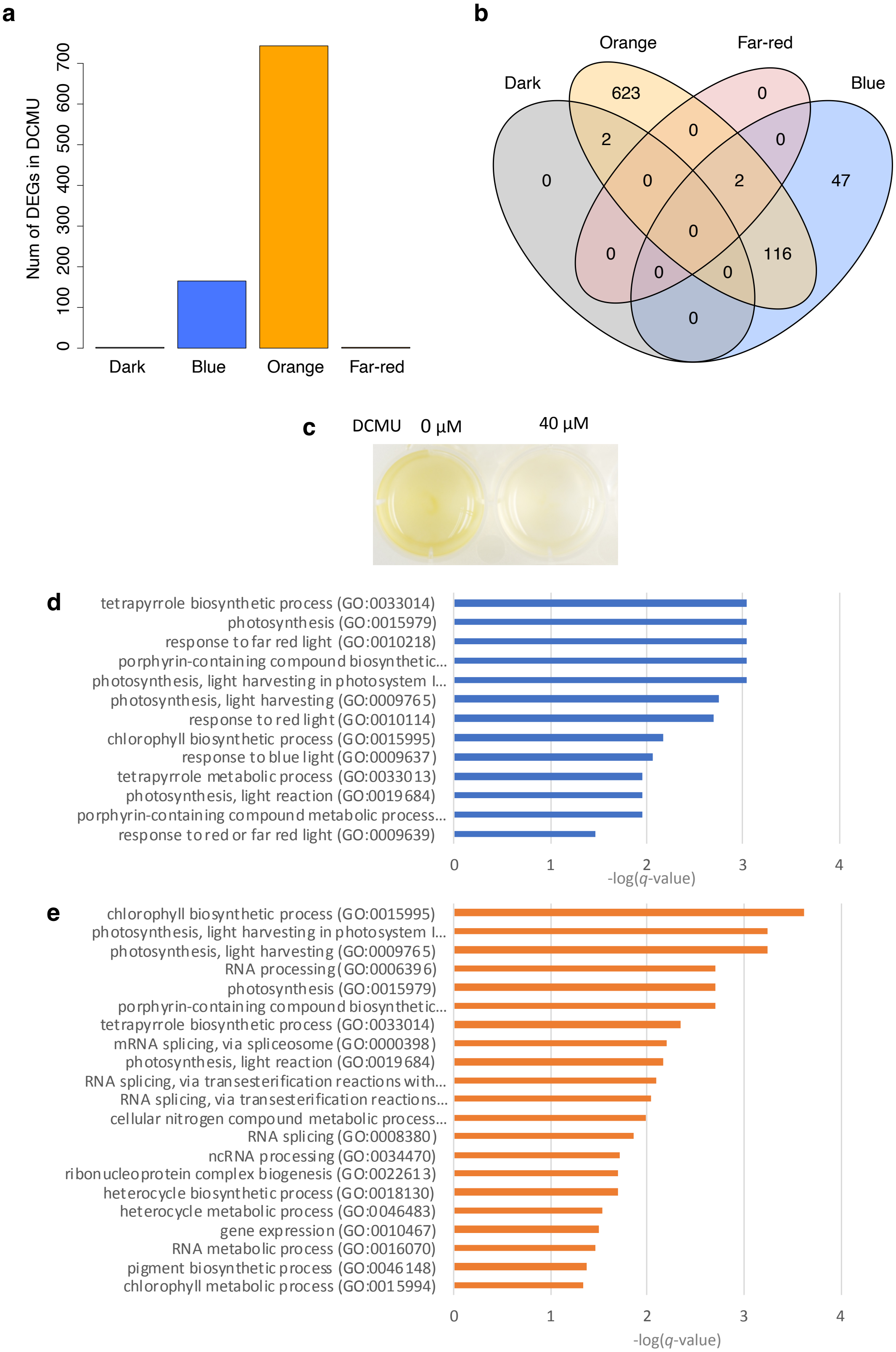
a
Num of DEGs in DCMU
0
100
200
300
400
500
600
700
Dark
Blue
Orange
Far-red
b
Orange
Far-red
Dark
Blue
623
0
2
0
0
0
0
2
47
0
0
0
116
0
0
0
c
DCMU
0 μM
40 μM
d
tetrapyrrole biosynthetic process (GO:0033014)
photosynthesis (GO:0015979)
response to far red light (GO:0010218)
porphyrin-containing compound biosynthetic...
photosynthesis, light harvesting in photosystem I...
photosynthesis, light harvesting (GO:0009765)
response to red light (GO:0010114)
chlorophyll biosynthetic process (GO:0015995)
response to blue light (GO:0009637)
tetrapyrrole metabolic process (GO:0033013)
photosynthesis, light reaction (GO:0019684)
porphyrin-containing compound metabolic process...
response to red or far red light (GO:0009639)
0
1
2
3
4
-log(q-value)
e
chlorophyll biosynthetic process (GO:0015995)
photosynthesis, light harvesting in photosystem I...
photosynthesis, light harvesting (GO:0009765)
RNA processing (GO:0006396)
photosynthesis (GO:0015979)
porphyrin-containing compound biosynthetic...
tetrapyrrole biosynthetic process (GO:0033014)
mRNA splicing, via spliceosome (GO:0000398)
photosynthesis, light reaction (GO:0019684)
RNA splicing, via transesterification reactions with...
RNA splicing, via transesterification reactions...
cellular nitrogen compound metabolic process...
RNA splicing (GO:0008380)
ncRNA processing (GO:0034470)
ribonucleoprotein complex biogenesis (GO:0022613)
heterocycle biosynthetic process (GO:0018130)
heterocycle metabolic process (GO:0046483)
gene expression (GO:0010467)
RNA metabolic process (GO:0016070)
pigment biosynthetic process (GO:0046148)
chlorophyll metabolic process (GO:0015994)
0
1
2
3
4
-log(q-value)

Supplementary Fig. 11. Effect of DCMU treatment on RNA-seq analysis.

(a) Number of DEGs between DCMU treated and untreated samples. A DEG is defined as a >1.5-fold for gene expression with a q-value < 0.05. **(b)** Venn diagram of DEGs in (a). **(c)** Effect of DCMU on *P. provasolii* cells. The cells were treated with 40 μM DCMU in 0.1% DMSO or only 0.1% DMSO (control) for 14 days. **(d)** GO enrichment analysis of 165 DEGs under blue light. **(e)** GO enrichment analysis of 743 DEGs under orange light.

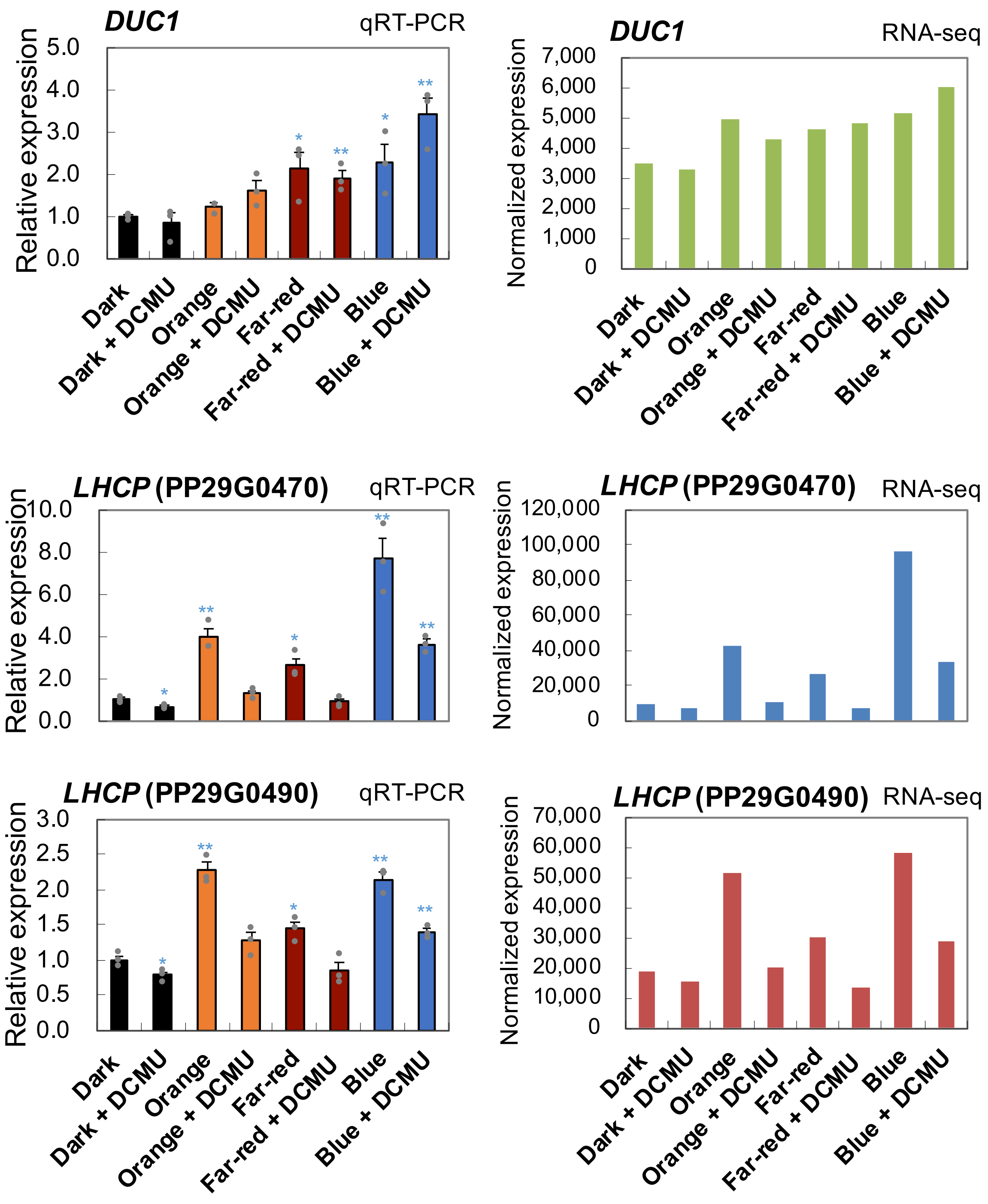

Supplementary Fig. 12. Quantitative RT-PCR and RNA-seq data comparison of the expression of *DUC1* and two *Lhcps.*

Quantitative RT-PCR analysis on the expression of the *DUC1* gene and *Lhsp* genes in *P. provasolii* exposed to monochromatic light after dark acclimatization (left). *P. provasolii* cells were treated with or without 40 µM DCMU. The transcript levels are presented as relative to dark‒ grown cells without DCMU treatment. Expression levels were normalized to the *ACT*

gene. We performed qRT-PCR experiment three times and all the experiments were run in triplicate (n=3 independent experiments). All data are expressed as the mean ± s.e.m. Asterisks indicate the *p*-value of two-sided Student's t-test; *p < 0.05, **p < 0.01 as relative to dark - grown cells without DCMU treatment. Normalized expression in the RNA-seq analysis is shown on the right. The expression of genes is presented as normalized read counts calculated with DEseq in R library. The source data of qRT-PCR are provided in the Source Data file.

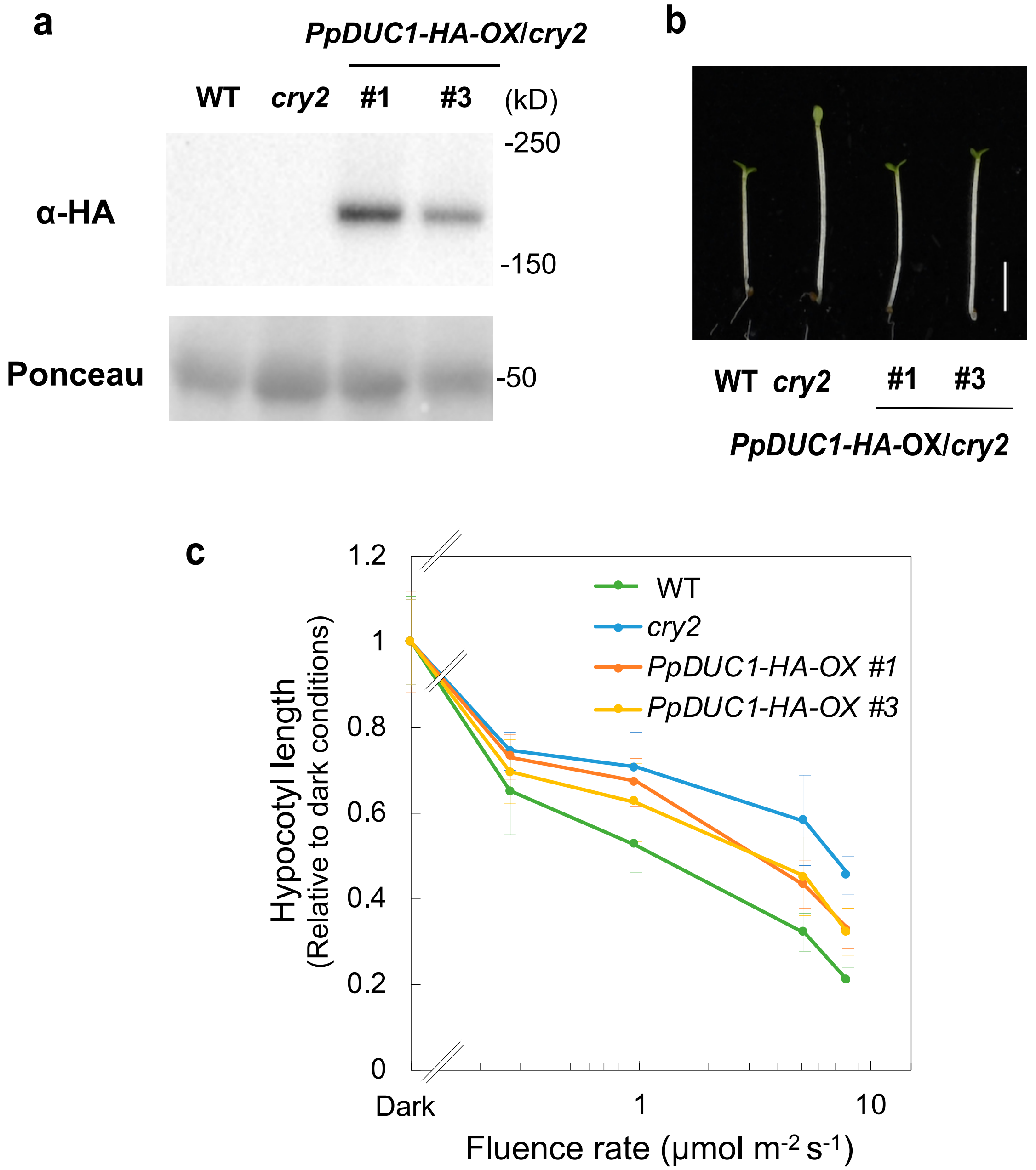

Supplementary Fig. 13. Expression of PpDUC1-HA and light fluence response curves for hypocotyl length of transgenic *Arabidopsis* in the *cry2* mutant background.

(a) Immunoblot analysis of total protein extract from 12-day-old seedlings of wild type (WT, col-4), a *cry2* mutant (*cry2-1*), and transgenic lines with *PpDUC1* overexpressed in the *cry2* mutant background, grown in continuous white light (40 μmol m^{-2} s^{-1}). Protein extracts were probed with anti-HA antibody (upper panel) and Ponceau S staining of the membrane served as a control for equal loading (lower panel). **(b)** Phenotypes of seven-day-old seedlings grown in continuous blue light (5.2 μmol m^{-2} s^{-1}). Scale bar = 1 mm. **(c)** Hypocotyl lengths of 7-day-old seedlings grown under continuous blue light with fluence rates of 0 to 8 μmol m^{-2} s^{-1} relative to

those grown under dark conditions. The results are presented as the means ± s.d. (n numbers of biologically independent samples are shown in source data in the Source Data file section). Similar results were obtained in two independent experiments. The source data of the immunoblot and hypocotyl length are provided in the Source Data file.

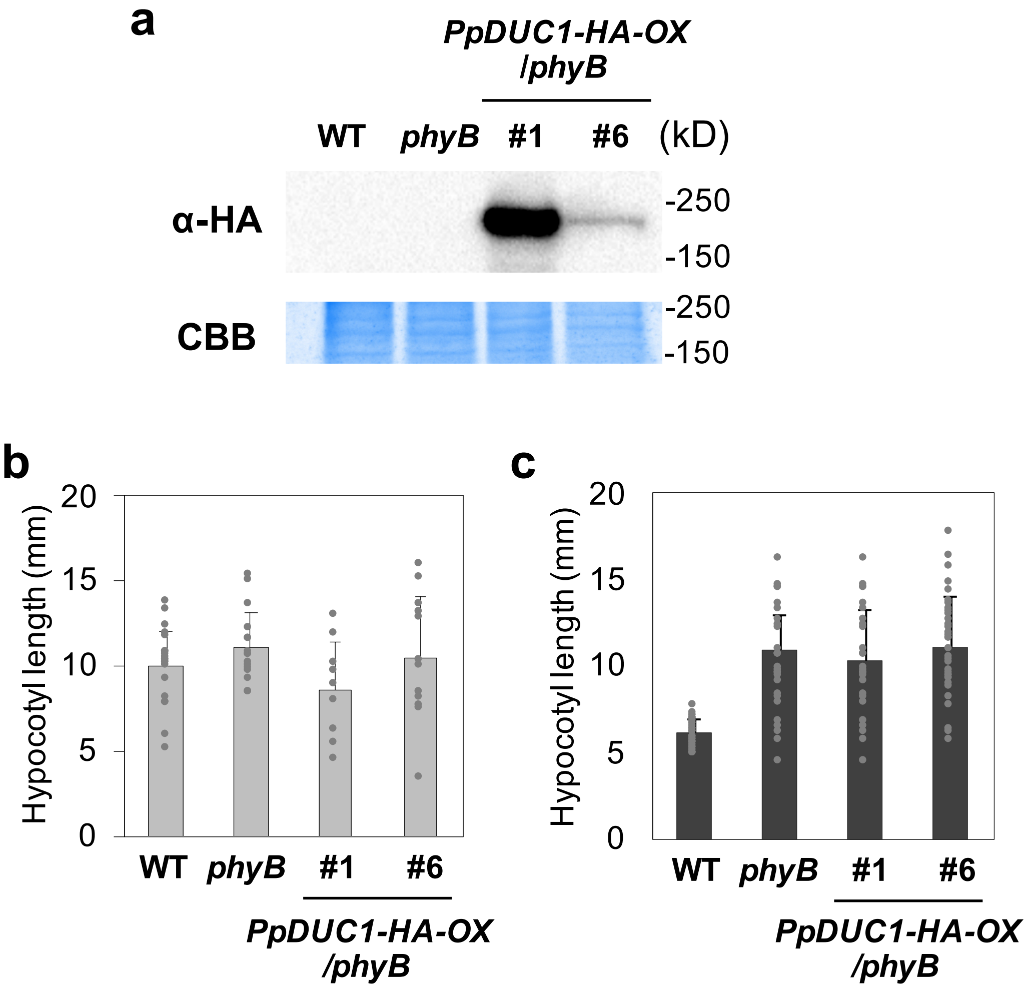

Supplementary Fig. 14. Expression of PpDUC1-HA and hypocotyl length of transgenic Arabidopsis in the *phyB* mutant background.

(a) Immunoblot analysis of total protein extract from 12-day-old seedlings of wild type (WT, col-0), a *phyB* mutant, and transgenic lines with PpDUC1 overexpressed in the *phyB* mutant background, grown in continuous white light (40 μmol m^{-2} s^{-1}). Protein extracts were probed with anti-HA antibody (upper panel) and Coomassie Brilliant Blue (CBB) staining of the gel served as a control for equal loading (lower panel). **(b)** Hypocotyl lengths of 7-day-old seedlings grown under continuous orange light of 1.3 μmol m^{-2} s^{-1}. As PpDUC1 was expected to be activated by light around the absorption maxima of PpPHY (624 nm) with PΦB, the phenotype under orange light (596 mm) was examined. The results are presented as the means ± s.d. (n=9 to 22). **(c)** Hypocotyl lengths of 7-day-old seedlings grown under continuous red light of 8.3 μmol m^{-2} s^{-1}. The results are presented as the means ± s.d. (n numbers of biologically independent samples are shown in source data in the Source Data file section). Similar results were obtained in two independent experiments. The source data of the immunoblot and hypocotyl length are provided in the Source Data file.

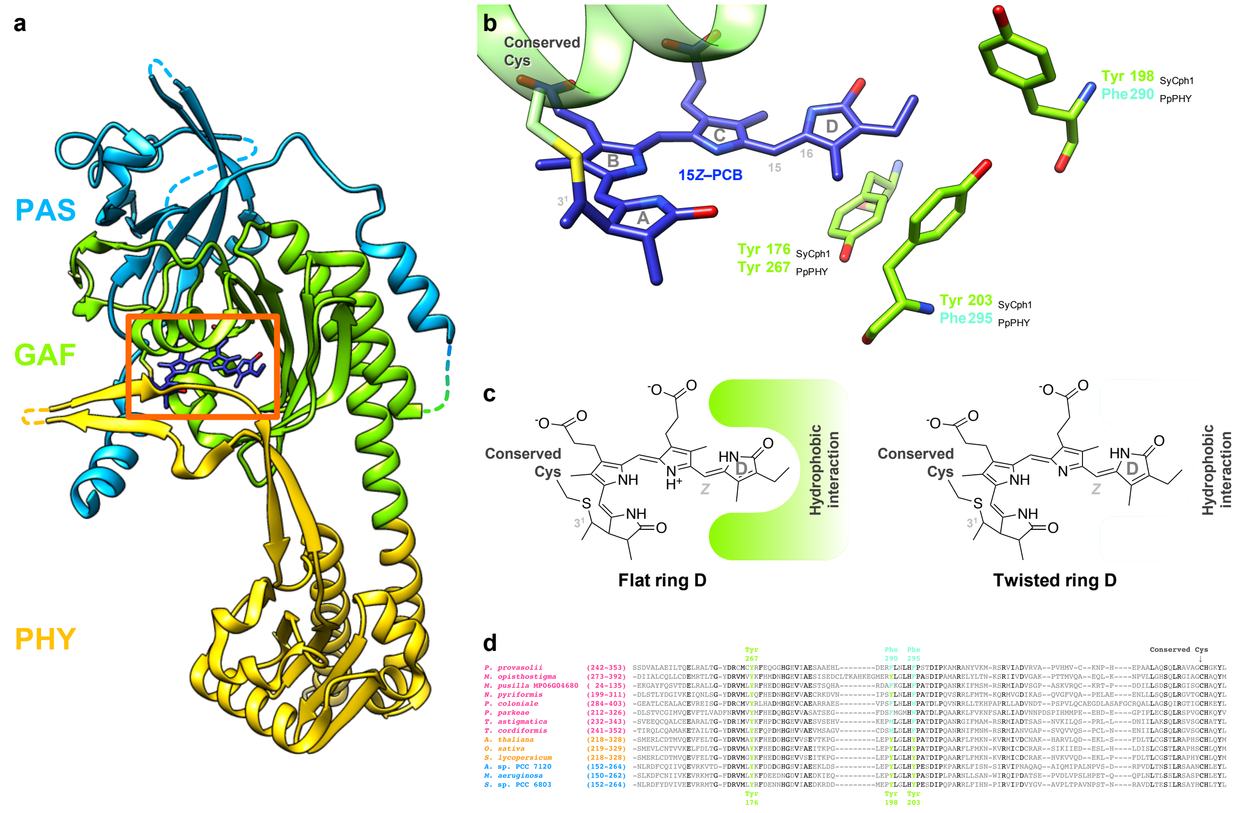

Supplementary Fig. 15. Potential color–tuning mechanism in phytochromes from prasinophytes.

(a) Whole structure of 15Z–PCB-bound SyCph1 (PDB ID: 2VEA)27 including PAS (blue), GAF (green) and PHY (yellow) domains. The chromophore (indigo blue) in the GAF domain is shown in the orange rectangle. **(b)** Possible amino acid residues involved with color-tuning. These candidate residues are near the chromophore (within 6Å) and highly conserved in phytochromes from land plants and cyanobacteria, but not in prasinophytes (shown in the alignment Supplementary Fig. 15d). Tyr176, Tyr198, and Tyr203 in SyCph1 are shown, corresponding to Tyr267, Phe290 and Phe295 in PpPHY. **(c)** Predicted models of color-tuning mechanisms in phytochromes from land plants and cyanobacteria (left), and from prasinophytes (right). Hydrophobic interaction between the chromophore and the candidate residues may affect the flat / twisted geometry of ring D, which is like the "trapped-twisted" mechanism of some cyanobacteriochromes. **(d)** Sequence alignment of phytochromes from prasinophytes (magenta), land plants (yellow) and cyanobacteria (cyan). The Tyr (lime green) and corresponding residues (such as Phe, Trp and Met residues, aqua blue), and highly conserved residues (black) are highlighted.

Supplementary Table 1. Spectral properties of PpPHY and PpCRY.

	**Chromophore**	**Dark state**	**Photoproduct state**	**Dark state – Photoproduct state**	
		λ_{max} (nm)	λ_{max} (nm)	Positive (nm)	Negative (nm)
**PpPHY**	PCB	612	702	609	702
**PpCRY**	FAD	447	366, 399	447	367, 399

Supplementary Table 2. Primer list.

Name		Sequence
In vitro analysis		
pET28a vector	Fw	5’-GGATCCGAATTCGAGCTC-3’
	Rv	5’-CATATGGCTGCCGCGCGG-3’
PpPHY	Fw	5’-CGCGGCAGCCATATGCCGGTTCCGGTACCAGCC-3’
	Rv	5’-CTCGAATTCGGATCCTTACTGCGCCACCAGCGAGCT-3’
PpCRY	Fw	5’-ATGCGAATTCCCACCAGACTCCGATGTGAGCGGCG-3’
	Rv	5’-GATCTCTAGACTACTGCGATCGATGGCGCTTCGCG-3’
In vivo analysis (*N. benthamiana)*		
PpDUC1	Fw	5’-ggggacaagtttgtacaaaaaagcaggcttcATGCCAGTGCCAGTGCCTGCTGC-3’
	Rv	5’-ggggaccactttgtacaagaaagctgggtcCTGCGATCGATGGCGCTTCGC-3’
PpPHY	Fw	5’-ggggacaagtttgtacaaaaaagcaggcttcATGCCAGTGCCAGTGCCTGCTGC-3’
	Rv	5’-ggggaccactttgtacaagaaagctgggtcAGCGTATGGCATGCACTGAAC-3’
PpCRY	Fw	5’-ggggacaagtttgtacaaaaaagcaggcttcATGCCACCAGACTCCGATGTGAGCGGCG-3’
	Rv	5’-ggggaccactttgtacaagaaagctgggtcCTGCGATCGATGGCGCTTCGC-3’
XbaI-*HY5*	Fw	5’-GGGACTCTAGAATGCAGGAACAAGCGACTAGCTCTTTAG-3’
In vivo analysis (*A. thaliana*)		
PpDUC1-HA	Fw	5’-CACCATGCCAGTGCCAGTGCCTG-3’
	Rv	5’-CTAAGCATAATCTGGTACATCATATGGATACTGCGATCGATGGCGCTTCGC-3’
qRT-PCR analysis		
PpDUC1	Fw	5’-GTTCCCTGGAACTACGACCA-3’
	Rv	5’-TCCCAGAAGTGGTGAAGACC-3’
Lhcp (*PP29G0470*)	Fw	5’-CATCTTCGAGGAGCTCAAGG-3’
	Rv	5’-ACACACCAACCGCTTACGAC-3’
Lhcp (*PP29G0490*)	Fw	5’-CATCTTCGAGGAGCTCAAGG-3’
	Rv	5’-ACGCAATGAGACACTTCACG-3’
ACT	Fw	5’-ACGCAATGAGACACTTCACG-3’
	Rv	5’-TCCACATCTGCTGAAACGTC-3’

Restriction sites on each primer are indicated by underlining.
Uppercase letters indicate sequences from genomic DNA.
Lowercase letters indicate adaptor sequences for the Gateway system.
Fw: Forward primer, Rv: Reverse primer.

Supplementary references

1. Blanc-Mathieu, R. *et al.* An improved genome of the model marine alga *Ostreococcus tauri* unfolds by assessing Illumina de novo assemblies. *BMC Genomics* **15**, 1103 (2014).
2. Blanc-Mathieu, R. *et al.* Population genomics of picophytoplankton unveils novel chromosome hypervariability. *Sci Adv* **3**, e1700239 (2017).
3. Palenik, B. *et al.* The tiny eukaryote Ostreococcus provides genomic insights into the paradox of plankton speciation. *Proc Natl Acad Sci USA* **104**, 7705–7710 (2007).
4. Yau, S. *et al.* Virus-host coexistence in phytoplankton through the genomic lens. *Sci Adv* **6**, eaay2587 (2020).
5. Worden, A. Z. *et al.* Green evolution and dynamic adaptations revealed by genomes of the marine picoeukaryotes Micromonas. *Science* **324**, 268–272 (2009).
6. Moreau, H. *et al.* Gene functionalities and genome structure in *Bathycoccus prasinos* reflect cellular specializations at the base of the green lineage. *Genome Biol.* **13**, R74 (2012).
7. Burns, J. A., Paasch, A., Narechania, A. & Kim, E. Comparative genomics of a bacterivorous green alga reveals evolutionary causalities and consequences of phago-mixotrophic mode of nutrition. *Genome Biol. Evol.* **7**, 3047–3061 (2015).
8. Lemieux, C., Turmel, M., Otis, C. & Pombert, J.-F. A streamlined and predominantly diploid genome in the tiny marine green alga *Chloropicon primus*. *Nat. Commun.* **10**, 4061 (2019).
9. Junkins, E. N. *et al.* Draft genome sequence of Picocystis sp. strain ML, Cultivated from Mono Lake, California. *Microbiol Resour Announc* **8**, (2019).
10. Tyler, C. R. S. *et al.* High-quality draft genome sequence of the green alga *Tetraselmis*

striata (Chlorophyta) generated from PacBio sequencing. *Microbiology Resource Announcements* **8** (2019).

11. Hoffmann, S. *et al.* A multi-split mapping algorithm for circular RNA, splicing, trans-splicing and fusion detection. *Genome Biol.* **15**, R34 (2014).
12. Tanifuji, G., Erata, M., Ishida, K.-I., Onodera, N. & Hara, Y. Diversity of secondary endosymbiont-derived actin-coding genes in cryptomonads and their evolutionary implications. *Journal of Plant Research* **119**, 205–215 (2006).
13. Schneider, C. A., Rasband, W. S. & Eliceiri, K. W. NIH Image to ImageJ: 25 years of image analysis. *Nature Methods* **9**, 671–675 (2012).
14. Marçais, G. & Kingsford, C. A fast, lock-free approach for efficient parallel counting of occurrences of k-mers. *Bioinformatics* **27**, 764–770 (2011).
15. Grigoriev, I. V. *et al.* PhycoCosm, a comparative algal genomics resource. *Nucleic Acids Res.* **49**, D1004–D1011 (2021).
16. Keeling, P. J. *et al.* The Marine Microbial Eukaryote Transcriptome Sequencing Project (MMETSP): illuminating the functional diversity of eukaryotic life in the oceans through transcriptome sequencing. *PLoS Biol.* **12**, e1001889 (2014).
17. Katoh, K. & Toh, H. Recent developments in the MAFFT multiple sequence alignment program. *Brief. Bioinform.* **9**, 286–298 (2008).
18. Capella-Gutierrez, S., Silla-Martinez, J. M. & Gabaldon, T. trimAl: a tool for automated alignment trimming in large-scale phylogenetic analyses. *Bioinformatics* **25**, 1972–1973 (2009).
19. Darriba, D. *et al.* ModelTest-NG: A new and scalable rool for the selection of DNA and protein evolutionary models. *Mol. Biol. Evol.* **37**, 291–294 (2020).

20. Kozlov, A. M., Darriba, D., Flouri, T., Morel, B. & Stamatakis, A. RAxML-NG: a fast, scalable and user-friendly tool for maximum likelihood phylogenetic inference. *Bioinformatics* **35**, 4453–4455 (2019).

21. Emms, D. M. & Kelly, S. OrthoFinder: phylogenetic orthology inference for comparative genomics. *Genome Biol.* **20**, 238 (2019).

22. Nordberg, H. *et al.* The genome portal of the Department of Energy Joint Genome Institute: 2014 updates. *Nucleic Acids Res.* **42**, D26–31 (2014).

23. Nguyen, L.-T., Schmidt, H. A., von Haeseler, A. & Minh, B. Q. IQ-TREE: A Fast and Effective Stochastic Algorithm for Estimating Maximum-Likelihood Phylogenies. *Molecular Biology and Evolution* **32**, 268–274 (2015).

24. Curtis, M. D. & Grossniklaus, U. A gateway cloning vector set for high-throughput functional analysis of genes in planta. *Plant Physiol.* **133**, 462–469 (2003).

25. Guo, H., Yang, H., Mockler, T. C. & Lin, C. Regulation of flowering time by Arabidopsis photoreceptors. *Science* **279**, 1360–1363 (1998).

26. Clough, S. J. & Bent, A. F. Floral dip: a simplified method for *Agrobacterium*-mediated transformation of *Arabidopsis thaliana*. *The Plant Journal* **16**, 735–743 (1998).

27. Essen, L. O., Mailliet, J. & Hughes, J. The structure of a complete phytochrome sensory module in the Pr ground state. *Proc Natl Acad Sci USA.* **105**, 14709–14714 (2008).

28. Pettersen, E. F. *et al.* UCSF Chimera--a visualization system for exploratory research and analysis. *J. Comput. Chem.* **25**, 1605–1612 (2004).

29. Rockwell, N. C. & Lagarias, J. C. Phytochrome evolution in 3D: deletion, duplication, and diversification. *New Phytol.* **225**, 2283–2300 (2020).

30. Fushimi, K. *et al.* Photoconversion and fluorescence properties of a red/green-type

cyanobacteriochrome AM1_C0023g2 that binds not only phycocyanobilin but also biliverdin. *Front. Microbiol.* **7**, 588 (2016).

31. Narikawa, R. *et al.* A biliverdin-binding cyanobacteriochrome from the chlorophyll d-bearing cyanobacterium Acaryochloris marina. *Sci. Rep.* **5**, 7950 (2015).
32. Liu, B., Liu, H., Zhong, D. & Lin, C. Searching for a photocycle of the cryptochrome photoreceptors. *Curr. Opin. Plant Biol.* **13**, 578–586 (2010).
