# Supplementary material for: Identification of a dual orange/far-red and blue light photoreceptor from an oceanic green picoplankton
Source: Nat Commun. 2021 Jun 16;12:3593. doi: 10.1038/s41467-021-23741-5 (PMC8209157; doi:10.1038/s41467-021-23741-5)
Supplement: Supplementary file 3 — Reporting Summary [file 41467_2021_23741_MOESM3_ESM.pdf]

nature research

Corresponding author(s): Matsui Minami

Last updated by author(s): May 8, 2021

Reporting Summary

Nature Research wishes to improve the reproducibility of the work that we publish. This form provides structure for consistency and transparency in reporting. For further information on Nature Research policies, see our Editorial Policies and the Editorial Policy Checklist.

Statistics

For all statistical analyses, confirm that the following items are present in the figure legend, table legend, main text, or Methods section.

n/a	Confirmed	
☐	☒	The exact sample size (*n*) for each experimental group/condition, given as a discrete number and unit of measurement
☐	☒	A statement on whether measurements were taken from distinct samples or whether the same sample was measured repeatedly
☐	☒	The statistical test(s) used AND whether they are one- or two-sided<br>*Only common tests should be described solely by name; describe more complex techniques in the Methods section.*
☒	☐	A description of all covariates tested
☐	☒	A description of any assumptions or corrections, such as tests of normality and adjustment for multiple comparisons
☐	☒	A full description of the statistical parameters including central tendency (e.g. means) or other basic estimates (e.g. regression coefficient) AND variation (e.g. standard deviation) or associated estimates of uncertainty (e.g. confidence intervals)
☐	☒	For null hypothesis testing, the test statistic (e.g. *F*, *t*, *r*) with confidence intervals, effect sizes, degrees of freedom and *P* value noted<br>*Give P values as exact values whenever suitable.*
☐	☒	For Bayesian analysis, information on the choice of priors and Markov chain Monte Carlo settings
☒	☐	For hierarchical and complex designs, identification of the appropriate level for tests and full reporting of outcomes
☐	☒	Estimates of effect sizes (e.g. Cohen's *d*, Pearson's *r*), indicating how they were calculated

Our web collection on statistics for biologists contains articles on many of the points above.

Software and code

Policy information about availability of computer code

Data collection: Publicly available data were downloaded and no specific software was applied.

Data analysis:

-Genome sequencing
Genome size estimation: Jellyfish 2.1.4 [https://github.com/gmarcais/Jellyfish]
Quality trimming of illumina reads: Trimmomatic version 0.36 [https://github.com/usadellab/Trimmomatic]
Error correction of nanopore reads: CONSENT v1.1.2 [https://github.com/morispi/CONSENT]
Assembler: MaSuRCA 3.3.2 [https://github.com/alekseyzimin/masurca]
Scaffolding tool: Fast-SG [https://github.com/adigenova/fast-sg]
Gap-filling tool: LR_Gapcloser v1.1 [https://github.com/CAFS-bioinformatics/LR_Gapcloser]
Mapping tool: BWA version 0.7.17 [https://github.com/lh3/bwa]
Polishing tool: Pilon 1.22 [https://github.com/broadinstitute/pilon]

-Genome annotation
Gene model construction and functional annotation pipeline: funannotate v1.5.3 [https://github.com/nextgenusfs/funannotate]
Functional annotation: eggNOG web server v1 [http://eggnog-mapper.embl.de/]
Mapping RNA-seq reads to CDSs: minimap2 2.17 [https://github.com/lh3/minimap2]
Quality check of genome completeness: BUSCO v2/v32 via gVolante [https://gvolante.riken.jp/]

-PFGE
ImageJ 1.52q [https://imagej.nih.gov/ij/]

- RNA-seq
Trimming the low quality reads: FASTX-Toolkit-0.0.14 [http://hannonlab.cshl.edu/fastx_toolkit/index.html]

Mapping of RNA-seq reads: STAR v2.5.0c [https://github.com/alexdobin/STAR]
Normalized of read counts: R package DEseq 1.42.0 [https://bioconductor.org/packages/release/bioc/html/DESeq.html]

- Domain search
InterProScan v5.40-77.0 [https://www.ebi.ac.uk/interpro/download/]
SMART [http://smart.embl-heidelberg.de/]

-Phylogenetic analysis
Alignment tool: MAFFT v7.453/v7.427 [https://mafft.cbrc.jp/alignment/software/]
Trimming of alignment: trimAl v1..4.rev15 [http://trimal.cgenomics.org/]
Model test: ModelTest-NG v0.1.5 [https://github.com/ddarriba/modeltest]
ML analysis: RAxML-NG v0.9.0 [https://github.com/amkozlov/raxml-ng]
ML analysis: IQ-TREE v1.6.12 [http://www.iqtree.org/]
Bayesian analysis: MrBayes v3.2.7a [https://nbisweden.github.io/MrBayes/index.html]

-Metagenomic analysis
Mapping tool: segemehl 0.3.4 [https://www.bioinf.uni-leipzig.de/Software/segemehl/]

-Genome comparison
Detecting orthogroups: OrthoFinder version 2..3.10 [https://github.com/davidemms/OrthoFinder]

-Analyses of read coverage and structural variants
Repeat Masking: RepeatModeler 1.0.11 [https://github.com/Dfam-consortium/RepeatModeler]
Repeat Masking: RepeatMasker 4.1.0 [https://www.repeatmasker.org/]
Mapping tool: minimap2 2.17 [https://github.com/lh3/minimap2]
Detection of SNPs and read coverage: get_SNP.pl in SSRG pipeline [https://github.com/PombertLab]
Detection of SNPs: VarScan v2.4.4 [http://varscan.sourceforge.net/]

For manuscripts utilizing custom algorithms or software that are central to the research but not yet described in published literature, software must be made available to editors and reviewers. We strongly encourage code deposition in a community repository (e.g. GitHub). See the Nature Research guidelines for submitting code & software for further information.

Data

Policy information about availability of data

All manuscripts must include a data availability statement. This statement should provide the following information, where applicable:

- Accession codes, unique identifiers, or web links for publicly available datasets
- A list of figures that have associated raw data
- A description of any restrictions on data availability

The genome and transcriptome data are deposited in the DDBJ/EMBL/GenBank under the accession number of GCA_015473125.1 [https://www.ncbi.nlm.nih.gov/assembly/GCA_015473125.1/], PRJDB10693 [https://www.ncbi.nlm.nih.gov/bioproject/?term=PRJDB10693], respectively.
The genome browser and transcriptome data of P. provasolii are available at: http://matsui-lab.riken.jp/JBrowse/index.html?data=data%2Fpycnococcus.

The publicly available datasets in this study are listed here: PhycoCosm database [https://phycocosm.jgi.doe.gov/phycocosm/home], Ocean Monitoring Database of Sendai Bay and the western subarctic Pacific Ocean [http://marine-meta.healthscience.sci.waseda.ac.jp/crest/metacrest/graphs/],
MMETSP (The Marine Microbial Eukaryote Transcriptome Sequence Project) [https://www.imicrobe.us/#/projects/104]
Source data are provided with this paper.

Field-specific reporting

Please select the one below that is the best fit for your research. If you are not sure, read the appropriate sections before making your selection.

☒ Life sciences ☐ Behavioural & social sciences ☐ Ecological, evolutionary & environmental sciences

For a reference copy of the document with all sections, see nature.com/documents/nr-reporting-summary-flat.pdf

Life sciences study design

All studies must disclose on these points even when the disclosure is negative.

Sample size

For biological data such as hypocotyl length, sample size were empirically selected during experimental design and details were described in the figure legends. No sample size calculation was performed.

For all the phylogenetic analyses, non-parametric bootstrap analyses (BP) were replicated at least 100 times. It is likely to be empirically sufficient, but no sample size calculation was performed. For Bayesian analysis, the inference consisted of 1,000000 generations with sampling every 1,000 generations using four MCMCMC simulations. Two separate runs were performed, and Bayesian posterior probabilities (BPP) were calculated from the majority rule consensus of the tree sampled after the initial 250 burn-in trees. We confirmed sufficient sampling size by checking average standard deviation of split frequencies (ASDSF) < 0.01.

Data exclusions

No data was excluded from the manuscript.

Replication	Experiments have been done at least two times to successfully verify replications.
Randomization	All samples were arranged ramdomly into experimental groups.
Blinding	Not relevant to this study, because the different lines show clear phenotypes compared with the wild type. Blinding was not possible becouse experimenters had knowledge of mutants.<br><br>It is not applicable for genome and transcriptome analysis.

Reporting for specific materials, systems and methods

We require information from authors about some types of materials, experimental systems and methods used in many studies. Here, indicate whether each material, system or method listed is relevant to your study. If you are not sure if a list item applies to your research, read the appropriate section before selecting a response.

Materials & experimental systems

n/a	Involved in the study	
☐	☒	Antibodies
☒	☐	Eukaryotic cell lines
☒	☐	Palaeontology and archaeology
☒	☐	Animals and other organisms
☒	☐	Human research participants
☒	☐	Clinical data
☒	☐	Dual use research of concern

Methods

n/a	Involved in the study	
☒	☐	ChIP-seq
☒	☐	Flow cytometry
☒	☐	MRI-based neuroimaging

Antibodies

Antibodies used	Anti-GFP antibody; Torrey Pines Biolabs Inc, TP401, AmershamTM protein A horseradish peroxidase linked antibody; GE Healthcare Corp., NA9120, Anti-HA-Peroxidase, High Affinity from rat IgG1; Roshe 12013819001
Validation	We examined its specific recognition by using E.coli expressed GFP through protein-blot assay. https://www.labome.com/product/Torrey-Pines-Biolabs/TP401.html<br>Protein A HRP linked antibody, https://www.citeab.com/antibodies/3288278-na935-amersham-ecl-rat-igg-hrp-linked-whole-antibod<br>https://www.sigmaaldrich.com/content/dam/sigma-aldrich/docs/Roche/Bulletin/1/12013819001bul.pdf
